# Mechanical and Microstructural Performance of Different Plaster Mortars Used to Protect Interior Concrete Exposed to Elevated Temperatures

**DOI:** 10.3390/polym18172105

**Published:** 2026-08-29

**Authors:** İbrahim Türkmen, Muhammed Şamil Gürkan, Enes Ekinci, Ramazan Demirboğa, Abdulrahman Ahmed Alymani

**Affiliations:** 1 Department of Architectural Engineering, College of Engineering and Advanced Computing, Alfaisal University, Riyadh 11533, Saudi Arabia; iturkmen@alfaisal.edu (İ.T.); rdemirboga@alfaisal.edu (R.D.); abalymani@alfaisal.edu (A.A.A.); 2 Department of Civil Engineering, Inonu University, Malatya 44280, Türkiye; enes.ekinci@inonu.edu.tr; 3 Department of Construction, Hekimhan Mehmet Emin Sungur Vocational School, Malatya Turgut Ozal University, Malatya 44400, Türkiye

**Keywords:** elevated temperature, geopolymer, calcium aluminate cement, plaster mortar, thermal protection of concrete

## Abstract

The physical, mechanical, and microstructural degradation that occurs in concrete elements exposed to high temperatures constitutes an important research topic in terms of the fire safety and service performance of structures. In this study, the behavior of plaster mortars produced with cement-based and ground granulated blast furnace slag (GGBFS)-based binders, as well as normal concrete (interior concrete) specimens coated with these mortars, under high-temperature exposure was experimentally investigated. After the prepared mortars and plastered concrete specimens were exposed to temperatures of 100, 300, 500, and 700 °C, changes in compressive strength, ultrasonic pulse velocity (UPV), water absorption, mass loss, and bulk density were evaluated. Analysis of the experimental results showed that high temperatures, particularly 300 °C and above, caused significant performance losses in all binder systems. Although calcium aluminate cement-based mortars developed high early-age strength, they exhibited more pronounced strength losses under elevated temperatures, whereas geopolymer-based binders demonstrated more stable performance at low and medium temperatures. Additionally, a strong correlation (R^2^ ≈ 0.89) was observed between UPV and compressive strength in the plastered interior concrete specimens. At 500 and 700 °C, the plastered concrete specimens exhibited 9.8–13.3% and 10.4–18.1% higher residual compressive strength, respectively, compared with the unplastered control specimens. At 700 °C, the PC-based plaster provided the highest improvement in residual compressive strength (18.1%), while the G-Na system exhibited the lowest water absorption, which was 19.5% lower than that of the control concrete. Plastered concrete specimens retained their mechanical and physical properties better than the control (unplastered) concrete specimens at all temperature levels, and this finding was further supported by microstructural analyses. The results indicate that plaster systems produced with different binders are effective in limiting thermal damage to the interior concrete.

## 1. Introduction

Concrete is one of the most widely used construction materials in infrastructure and buildings due to its high compressive strength, durability, cost-effectiveness, and availability of raw materials [1,2,3,4,5]. Despite these advantages, the inherent brittleness and durability-related limitations of concrete can affect the long-term reliability and structural safety of concrete structures [6]. Population growth and land constraints are making high-rise buildings and underground construction necessary, which in turn makes fire safety an even more critical issue. Fires in such structures in recent years have posed serious risks in terms of loss of life and property. Although concrete is known as a durable material, its microstructure degrades at high temperatures, its strength decreases, and damage such as cracking or spalling may occur [7,8,9,10]. This situation leads to the exposure of the reinforcing steel and a weakening of structural integrity. For this reason, the behavior of concrete under fire is an important area of research in terms of structural safety, and the effects of temperature are being carefully examined from both durability and safety perspectives. Consequently, the selection of construction materials for resistance to high temperatures is of the utmost importance [11,12,13,14,15].

Geopolymer- and alkali-activated materials have emerged as promising alternatives to conventional Portland cement-based materials, particularly because of their potential environmental and durability advantages. Alkali-activated slag (AAS) materials, for example, have been increasingly investigated as alternative construction materials due to their potential for utilizing industrial by-products and reducing the environmental impact associated with conventional cement production [16]. Geopolymer-based materials have also attracted considerable attention because of their favorable mechanical properties, durability, and potential resistance to elevated temperatures. With these properties, geopolymers are considered promising alternatives to traditional concrete in the construction industry, as they utilize industrial by-products, consume less energy, and feature low CO_2_ emissions [17,18,19,20,21]. In recent years, research on alkali-activated mortars, commonly referred to as “green concrete,” has increased rapidly as researchers have sought alternative binder systems for cement. In this context, geopolymer and alkali-activated materials have been increasingly studied for applications requiring adequate mechanical performance, durability, microstructural stability, and resistance to elevated temperatures [22,23,24,25,26].

As cement paste is exposed to increasing temperatures, various physicochemical changes are observed in its components [27]. At approximately 100 °C, the evaporation of bound water begins, and at around 180 °C, the dehydration of calcium silicate hydrate (C-S-H) occurs. When the temperature reaches 500 °C, calcium hydroxide decomposes, and at approximately 700 °C, further decomposition of calcium silicate hydrate occurs. These processes cause significant deterioration in the internal structure of the cement paste, leading to a gradual weakening of its mechanical properties. These chemical decompositions, which occur particularly when the temperature exceeds 500 °C, significantly accelerate the material’s loss of strength [28,29,30,31,32].

Geopolymers are amorphous aluminosilicate binders formed by the activation of industrial by-products rich in silica and alumina (fly ash, blast furnace slag, waste glass, etc.) using alkaline solutions such as sodium hydroxide, potassium hydroxide, or sodium silicate. Through this activation process, the materials form a three-dimensional Si–O–Al network structure and transform into a gel. The resulting structure exhibits high compressive strength, fire resistance, chemical resistance, and durability [33,34,35,36]. Geopolymer materials exhibit high thermal stability, low degradation, and superior fire resistance; therefore, they are a promising option for fire-resistant and sustainable building materials [37]. Alkali-activated slag systems with high calcium content promote the formation of a dense calcium–aluminum–silicate hydrate (C-A-S-H) gel, enabling rapid setting at ambient temperature [38]. However, at temperatures of 600 °C and above, the dehydration and decomposition of calcium-rich phases cause a significant decrease in mechanical strength; furthermore, high slag content further exacerbates mass loss and shrinkage resulting from thermal exposure [39,40]. GGBFS is a by-product of steel production and is widely used in geopolymer production due to its high aluminum, silica, and calcium content; however, its calcium-rich composition makes it more sensitive to heat, as the matrix begins to degrade by losing water when exposed to high temperatures [41].

Calcium aluminate cement (CAC) has attracted attention due to its exceptional properties and has become the binder of choice for applications such as refractory materials, industrial furnaces, and construction materials exposed to high temperatures. Rapid setting and high resistance to chemical corrosion are among CAC’s standout properties. In CAC production, carbon dioxide emissions can be significantly reduced thanks to the use of suitable raw materials and the lower sintering temperatures compared to Portland cement; for this reason, CAC is considered an environmentally friendly cement material [42]. The primary issue associated with CAC is that the CAH_10_ and C_2_AH_8_ phases—which are considered metastable at room temperature during hydration—transform over time into the thermodynamically stable C_3_AH_6_ and AH_3_ phases; this transformation causes both a decline in strength from initially high early values and a reduction in chemical resistance accompanied by a sudden loss of strength [42,43,44,45,46]. CAC mortar can achieve the 28-day strength of Portland cement mortar in as little as 6 h. The long-term strength development of CAC-based composites depends on curing conditions due to the formation and transformation of metastable hydrates, which makes it difficult to predict the ultimate strength [46]. Strength initially rises rapidly to a high early-stage level due to the formation of CAH_10_ and C_2_AH_8_. It then decreases to a minimum during the conversion process to C_3_AH_6_ and AH_3_. Subsequently, it increases again with prolonged hydration [42]. During the transformation, the formation of stable hydrates with a denser structure increases porosity, which in turn leads to a significant loss of strength [45]. It is known that the hydration products formed during CAC hydration are highly sensitive to temperature changes, and the transformation process occurs more rapidly under high temperature and humidity conditions [43,45].

Previous studies have investigated the behavior of concrete-based materials under elevated temperatures, focusing on thermal degradation, residual mechanical properties, cracking, and spalling. These studies have also demonstrated that plaster and protective coating layers can delay temperature rise and heat penetration within concrete specimens [47,48]. In addition, studies on geopolymer-based structural elements have shown that protective coatings can enhance fire resistance and improve the load-carrying capacity of structural members after exposure to elevated temperatures [49]. However, most previous studies have primarily focused either on the protective effect of a specific coating on the concrete substrate or on the high-temperature behavior of geopolymer-based coating materials, while a comparative assessment of different cementitious and alkali-activated plaster mortars, both as individual materials and as protective layers on Portland cement-based concrete, remains limited. Therefore, the present study addresses this gap by comparatively evaluating the high-temperature performance of five plaster mortar systems with different binder types, as well as their effectiveness in protecting Portland cement-based concrete against elevated-temperature exposure. This study investigated the extent to which mortar mixtures produced with different types of binders can maintain their strength and other mechanical properties when exposed to high temperatures that structures may encounter. The binders comprised Portland composite cement (PC), lime–Portland composite cement (L-PC), calcium aluminate cement (CAC), and blast-furnace slag activated with NaOH or KOH (G-Na and G-K, respectively). The selection of these binder systems was intended to provide a comparative evaluation of different binder classes with distinct chemical compositions and hydration or reaction mechanisms under elevated-temperature exposure. PC and L-PC were selected to represent Portland cement-based systems and to examine the effect of lime incorporation, while CAC was included because of its distinct hydration characteristics and high early-age strength. G-Na and G-K were selected as alkali-activated GGBFS systems to compare the effects of NaOH- and KOH-based activation. The use of NaOH- and KOH-based activation enabled a direct comparison of the effects of the alkali cation (Na^+^ versus K^+^) on the mechanical behavior and thermal resistance of the geopolymer mortars. This selection allowed both the high-temperature performance of the plaster mortars themselves and their ability to protect the interior concrete to be evaluated comparatively. Plaster mortar specimens measuring 50 × 50 × 50 mm were produced from the prepared mortars. In addition, these mortars were applied to the entire surfaces of 100 × 100 × 100 mm standard Portland cement-based concrete specimens to form a 25 mm thick plaster layer. The prepared mortars and concrete specimens with plaster coatings of varying compositions were exposed to high temperatures of 100, 300, 500, and 700 °C for 1 h following curing periods of 7, 14, and 28 days. Following exposure to different temperatures, the specimens were examined through mechanical property testing, strength evaluations, visual inspections, and microstructural analyses; the high-temperature performance of different binder types was evaluated comparatively.

## 2. Materials and Methods

### 2.1. Materials

CEM I 42.5 R-type cement was used as a binder in the production of the interior concrete samples. In addition, five different plaster mortars were produced using different binder types. The binder types used in these plaster mortars were Portland composite cement (CEM II/A-M), hydrated lime (SLL), calcium aluminate cement (CAC), and GGBFS activated with NaOH or KOH. The produced plaster mortars were subsequently applied to the concrete specimens to protect them against elevated temperature effects. The chemical and physical characteristics of these binder materials are presented in Table 1.

In addition, sodium hydroxide (NaOH) and potassium hydroxide (KOH) were used as alkali activators in the production of geopolymer mortar samples applied as plaster. These activator solutions were prepared with tap water at a concentration of 10 M. Crushed stone aggregates with particle sizes of 0–4 mm and 4–8 mm were used in the production of the interior concrete samples. Furthermore, river aggregates with a size range of 0–4 mm were preferred in the plaster mortars prepared for coating the interior concrete samples.

**Figure 1:**
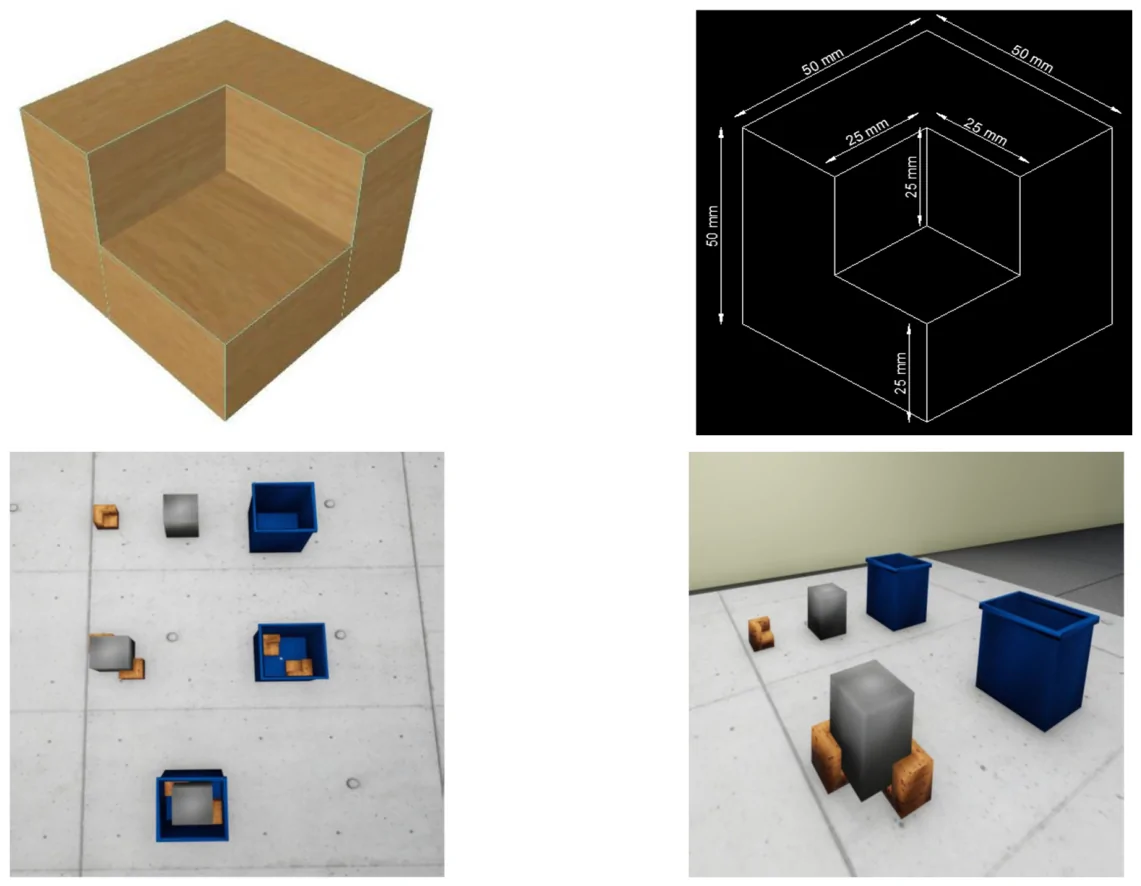
Schematic illustration of the geometry, dimensions, and centering stages of the wooden apparatus within the mould.

To obtain a uniform 25 mm plaster layer around the 100 × 100 × 100 mm concrete samples, wooden apparatuses were placed at two diagonally opposite lower corners of the 150 × 150 × 150 mm molds. This apparatus allowed reference concrete samples to be centered within the mold, creating a plaster layer of uniform thickness across all surfaces. ArchiCAD 12 and AutoCAD 2013 were used for the three-dimensional modeling and drafting, respectively, while Lumion 8.5 was used for rendering. The geometry, dimensions, and centering procedure of the wooden apparatus used in the specimen preparation are illustrated in Figure 1.

### 2.2. Experimental Procedure

#### 2.2.1. Mix Proportions

The interior concrete mixture was designed at a water-to-cement ratio of 0.43. The cement, fine-aggregate, and coarse-aggregate contents were 512, 614.02, and 878.85 kg/m^3^, respectively. For the plaster mortars, the binder-to-fine aggregate ratio was kept constant at 1:3 for all plaster types. For the geopolymer mixtures, NaOH and KOH solutions were prepared separately at a concentration of 10 M and allowed to cool to room temperature before being incorporated into the mixtures. The mixing ratios of the produced plasters are presented in Table 2, and the sample nomenclatures established according to the five different binder types are presented in Table 3.

#### 2.2.2. Preparation of Plastered Concrete Specimens

**Figure 2:**
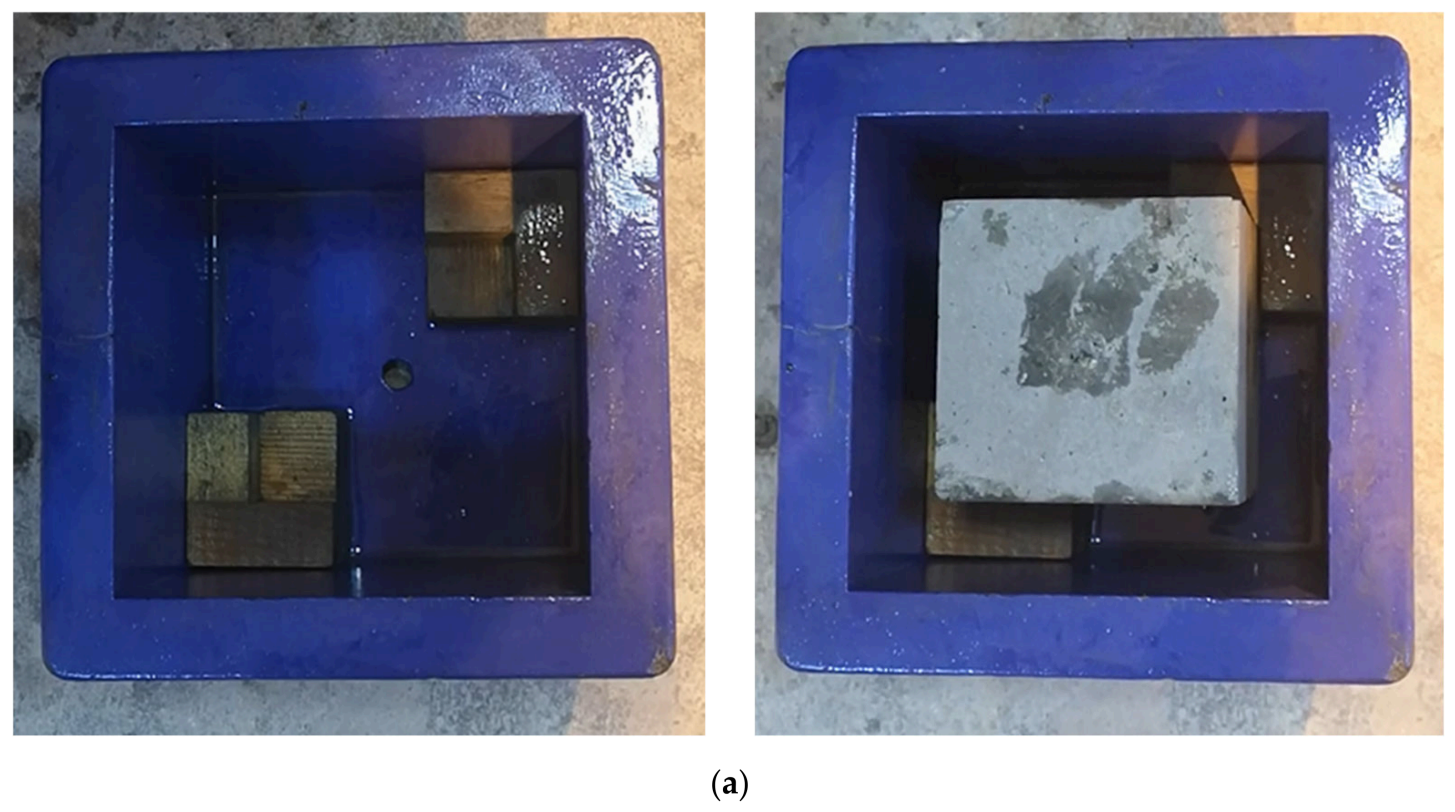

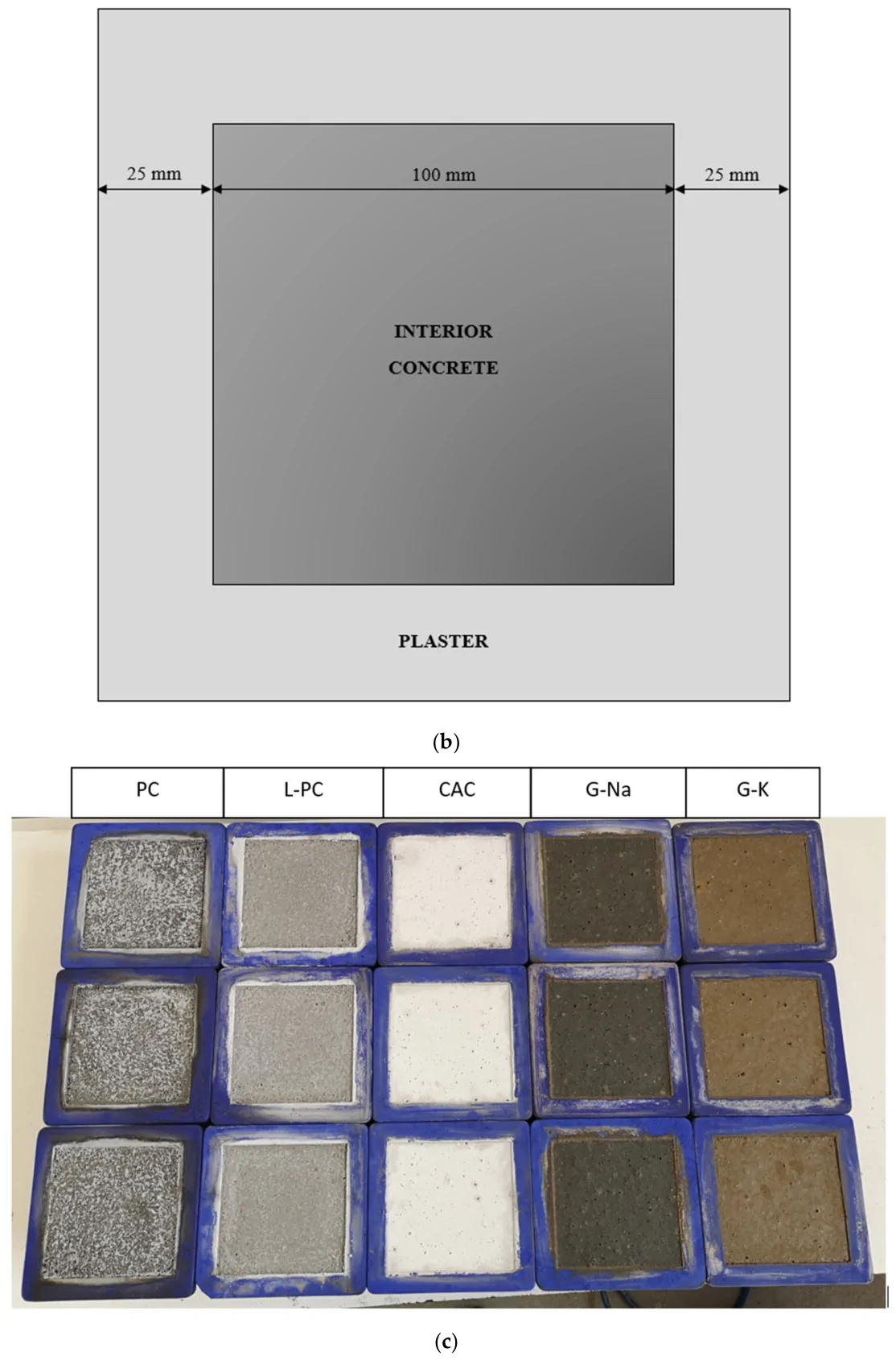
Preparation and geometry of the concrete specimens: (**a**) centering of the concrete cube specimen in the mold prior to plastering; (**b**) dimensioned schematic illustration of the plaster-coated concrete specimen; (**c**) appearance of the concrete specimens after coating with plaster mortar.

As seen in Figure 2a, wooden fixtures were placed at the diagonal lower corners of 150 × 150 × 150 mm plastic molds to allow 100 × 100 × 100 mm test concrete specimens to be coated with a 25 mm-thick layer of plaster mortar. These brackets ensured that a 25 mm gap remains on each side when the specimen is placed inside the mold, thereby securing the specimen in a centered position. After preparation of the interior concrete specimens, fresh plaster mortar was placed directly onto their surfaces within the mold to form a 25 mm-thick plaster layer. The plastered specimens were then cured under laboratory conditions for 7, 14, and 28 days. Figure 2b presents a dimensioned schematic illustration of the plaster-coated concrete specimen, showing the specimen dimensions and the 25 mm plaster thickness surrounding the interior concrete. The appearance of the plaster-coated interior concrete specimens in the molds is presented in Figure 2c.

### 2.3. Test Procedures

The tests were performed on the plaster mortar specimens after exposure to the respective temperatures and on the interior concrete specimens obtained from the plaster-coated concrete specimens after thermal exposure and removal of the plaster layer. Three specimens were prepared and tested at each temperature condition for each formulation, and the results are reported as mean values with standard deviations.

Compressive strength test: The compressive strength of the plaster mortars was determined in accordance with ASTM C109 [50] using 50 × 50 × 50 mm cube specimens. For the thermally exposed protected concrete specimens, the plaster layer was completely removed after thermal exposure, and the compressive strength was determined directly on the 100 × 100 × 100 mm concrete core. The 150 × 150 × 150 mm plaster-coated composite specimens were therefore not directly subjected to compression testing, and the load-bearing contribution of the plaster layer was not included in the measured strength. The concrete cores were loaded axially between two loading plates over a 100 × 100 mm loading area. The maximum load was recorded in kN, and the corresponding stress was obtained in N/mm^2^ (MPa). Accordingly, the measured values for the thermally exposed specimens represent the residual compressive strength of the concrete core protected by the plaster layer during thermal exposure. In addition, the compressive strengths of the plaster mortar and concrete specimens were determined at curing periods of 7, 14, and 28 days for each specimen group.

UPV measurement: The UPV values of the plaster mortars and interior concrete specimens were determined in accordance with the ASTM C597 standard [51].

Elevated temperature test: The plaster mortar specimens and interior concrete specimens were subjected to elevated temperatures of 100, 300, 500, and 700 °C. The plaster mortar specimens were kept under laboratory ambient conditions for 7, 14, and 28 days. The interior concrete specimens were coated with plaster mortar and kept under the same laboratory ambient conditions until the plaster layer had remained on the concrete for 7, 14, or 28 days before elevated-temperature exposure. Thermal exposure was performed using a high-temperature furnace with temperature monitoring and control. The specimens were placed in the furnace without contacting each other. The furnace temperature increased progressively, with approximate average heating rates of 12.5, 12.0, 4.5, and 2.5 °C/min corresponding to the target temperatures of 100, 300, 500, and 700 °C, respectively. Once the furnace reached the target temperature, the specimens were maintained at that temperature for 1 h. After the holding period, the specimens were removed from the furnace and allowed to cool naturally to room temperature under ambient conditions. After thermal exposure, the plaster layer was removed from the plaster-coated interior concrete specimens using a concrete cutting saw, and the remaining residues were removed with a spatula. No apparent damage to the interior concrete surface was observed during the removal process. The specimens were then subjected to the relevant tests to evaluate their residual properties after elevated-temperature exposure.

Water absorption test: The water absorption rate was determined on plaster mortar and interior concrete specimens in accordance with the ASTM C642 standard [52]. The water absorption properties of the samples were determined based on changes in mass. As part of the experiment, the samples were first dried in an oven (105 ± 5 °C) until they reached a constant mass; this value was recorded as the oven-dried mass (*A*). The samples were then immersed in water for a specific period until they were fully saturated, and their surfaces were wiped with a damp cloth; this value was recorded as the saturated surface dry mass (*B*).

The water absorption rate was calculated based on the mass increase using Equation (1) below:

(1) $$m=\left(\frac{B-A}{A}\right)\times 100$$

Here, m represents the sample’s water absorption rate by mass (%). The difference (*B* − *A*) represents the amount of water absorbed by the sample.

Mass loss test: Mass loss analysis was performed to determine the changes the produced samples underwent under the influence of high temperatures. In this context, the sample masses were measured before and after exposure to high temperatures using a balance with a precision of 1 g, and the percentage mass loss was calculated by dividing the mass difference by the initial mass. Equation (2) provides the formula for this calculation.

(2) $$W=\left(\frac{w_{i}-w_{s}}{w_{i}}\right)\times 100$$

To determine the changes in mass, the samples were weighed before ($w_{i}$) and after ($w_{s}$) exposure to high temperatures. The changes (W) were expressed as a percentage of the initial weights.

Bulk density: The determination of bulk density was performed on plaster mortar and interior concrete specimens in accordance with the ASTM C642 standard [52]. Because different types of binding materials were used in the mortar samples produced, the unit weight of the samples varied. The bulk density of the samples was calculated by dividing their dry weight by their volume.

Visual inspection: The effects of different temperatures on plaster mortars prepared with various types of binders were examined in terms of physical and visual changes. Cracks and color changes forming on the sample surfaces were observed, and the findings were evaluated for each sample group and supported by visual evidence.

Microstructural analysis: Samples were taken from interior concrete specimens that had been plastered with different types of mortar to determine their microstructural properties before and after exposure to high temperatures. The morphological structures of the collected samples were examined using a LEO-EVO 40 (Cambridge, UK) scanning electron microscope (SEM). In addition, X-ray diffraction (XRD) analyses were performed on powder samples obtained from the specimens to identify the phases present in the material. XRD analysis was performed using a Rigaku D/Max 2200 (Tokyo, Japan) diffractometer with Cu-Kα_1_ radiation (λ = 1.5405 Å) using the powder diffraction method. The diffraction patterns were collected over a 2θ range of 3–80° with a step size of 0.02°.

## 3. Results and Discussion

### 3.1. Compressive Strength

**Figure 3:**
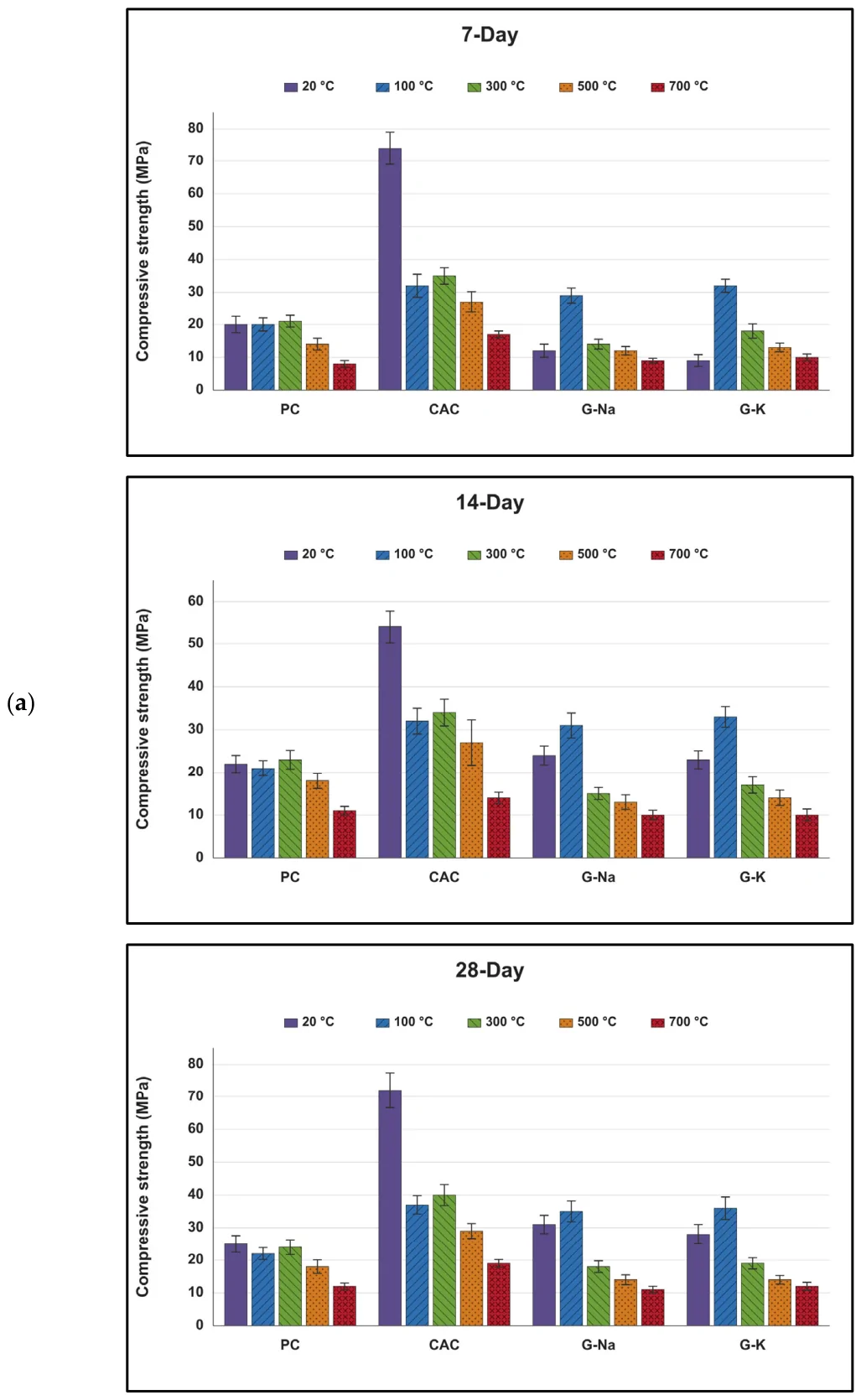

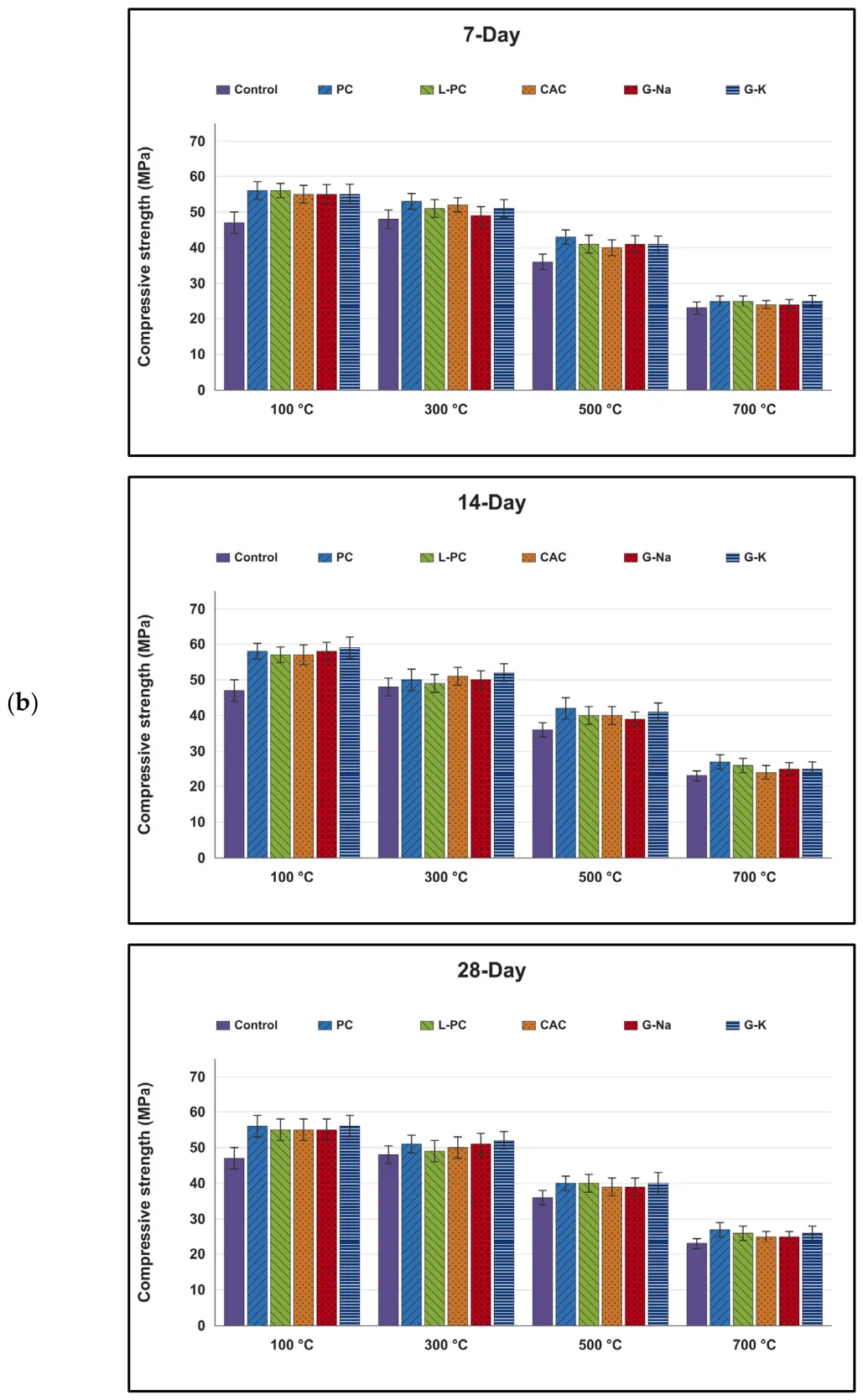
Compressive strength of (**a**) plaster-mortar specimens and (**b**) interior specimens at the reported curing ages and exposure temperatures.

Figure 3 shows how curing age and exposure temperature affected the compressive strength of the plaster mortars and plaster-coated concrete specimens after thermal exposure.

As shown in Figure 3a, the compressive strength results of plaster mortars produced with different types of binders showed that curing time and high temperature are decisive factors affecting mechanical performance. Due to low strength, the compressive strength value could not be measured for the L-PC group samples. In general, an increase in strength was observed across all binder systems as the curing time increased. For the 28-day specimens, increasing the exposure temperature from 20 °C to 700 °C resulted in compressive strength reductions ranging from 51.1% to 73.4% depending on the binder type.

In the PC group samples, a limited decrease in compressive strength was observed at 100 °C compared with the 20 °C control specimens, with a strength reduction of 11.7%. A partial recovery was observed at 300 °C, where the compressive strength increased by 6.9% compared with the value at 100 °C; however, it remained 5.6% lower than the 20 °C control specimens. Significant strength losses occurred at 500 °C and particularly at 700 °C, where the compressive strength reductions reached 25.8% and 51.1%, respectively, compared with the 20 °C control specimens. The slight decrease in compressive strength observed at 100 °C is due to the evaporation of free water within the specimen, which leads to the formation of pores and microcracks [53]. The increase in compressive strength observed around 300 °C is believed to be related to the formation of hydration products resulting from the acceleration of pozzolanic reactions as the temperature rises to 300 °C [54,55]. The loss of strength observed in samples exposed to a temperature of 500 °C is attributed to the dehydration of calcium hydroxide (Ca(OH)_2_) and the decomposition of the C–S–H gel [56,57]. At 700 °C, significant strength losses occurred due to the complete decomposition of CH and C–S–H [57,58].

CAC group specimens exhibited high early-age strength; however, they experienced significant strength losses under high-temperature exposure, which are attributed to transformation reactions. At 700 °C, the compressive strength decreased by 73.4% compared with the 20 °C control specimens. The rapid hydration of CAC releases a large amount of hydration heat within the first 24 h, resulting in higher early-age strength than OPC [59]. CAC blends achieve a significant portion of their final strength development during the first 7 days of the curing process [60]. This phenomenon is attributed to the rapid hydration of calcium aluminate minerals, which leads to the formation of calcium aluminate hydrates and an Al(OH)_3_ gel within a short period, thereby creating a strong early-age structure [61]. Yang et al. [62] determined that the compressive strength of 14-day-old specimens in high-alumina cement mortars was lower than that of 7-day-old specimens and attributed this to the transformation of the CAH_10_ and C_2_AH_8_ phases into the C_3_AH_6_ phase. However, they observed a renewed increase in the specimens’ strength after a 14-day curing period. The strength losses at high temperatures in the CAC group samples are attributed to the decomposition of hydration products, loss of bound water, and the development of pores and cracks resulting from the transformation of the CAH_10_ and C_2_AH_8_ phases into the C_3_AH_6_ phase [60,63].

In geopolymer systems (G-Na and G-K), exposure to 100 °C enhanced the compressive strength, with increases of 13.6% and 28.7% observed for G-Na and G-K specimens, respectively. This increase is attributed to additional gel formation and accelerated geopolymerization reactions [64,65]. However, strength losses occurred due to progressive structural degradation at temperatures of 300 °C and above [25,66,67]. The loss of compressive strength observed in geopolymer specimens following exposure to 500 °C is attributed to microcrack formation resulting from high thermal shrinkage caused by the evaporation of water within the geopolymer and thermal stresses generated by increased vapor pressure [68]. The loss of strength observed during exposure to a temperature of 700 °C is attributed to the decomposition of the calcium aluminosilicate hydrate (C-A-S-H) gel formed as a result of geopolymer hydration in an alkaline environment [69]. At 700 °C, G-K specimens exhibited a compressive strength loss of 56.9% compared with the 20 °C control values, whereas the corresponding loss for G-Na specimens was 64.7%. These results indicate that geopolymer systems activated with KOH exhibit superior high-temperature resistance compared with those activated with NaOH. This superior performance can be associated with the lower mass loss and volumetric shrinkage observed in K-based activator geopolymer systems, which can reduce thermally induced cracking during heating. The reduced formation of cracks limits stress concentration within the geopolymer matrix and consequently contributes to better retention of compressive strength under elevated temperatures [70]. Similarly, it has been reported in the literature that KOH-based geopolymers are more stable at high temperatures than NaOH-based geopolymers [71,72].

In general, it was determined that mechanical performance decreases in all bonding systems due to the effects of high temperatures; however, this decrease occurs to varying degrees depending on the type of binder. When the post-heating strength retention of plaster mortars was evaluated, the best performance at 700 °C was obtained for the PC system, which exhibited a compressive strength loss of 51.1% compared with the 20 °C reference condition. This was followed by the G-K, G-Na, and CAC systems, with strength losses of 56.9%, 64.7%, and 73.4%, respectively. However, the CAC system exhibited the highest absolute compressive strength values at all temperature levels due to its high initial strength, while it experienced a higher degree of strength loss at elevated temperatures due to the transformation and decomposition of hydration products.

As shown in Figure 3b, the protective effect of different plaster mortar compositions on the residual compressive strength of the interior concrete was evaluated after thermal exposure. At every exposure temperature, the plaster-coated concrete retained higher compressive strength than the unplastered control. Compared with the behavior observed at 300 °C, the protective effect of the plaster layer became more pronounced at higher temperatures. The residual compressive strengths of the plaster-coated specimens were found to be 9.8–13.3% higher at 500 °C and 10.4–18.1% higher at 700 °C compared with the corresponding uncoated control specimens. These findings suggest that the plaster layer provided a protective effect to the concrete core during high-temperature exposure, as reflected by the improved residual performance of the protected specimens. Depending on the binder type, geopolymer-based plasters exhibited higher protective performance at low and moderate temperatures (100–300 °C), with the G-K system providing the highest strength retention of 19.1% and 6.6%, respectively. In contrast, the PC-based plaster system demonstrated more effective protection at higher temperatures (500–700 °C). The PC system provided the highest protective performance at 700 °C, providing an 18.1% higher residual compressive strength compared with the uncoated control specimens exposed to the same temperature. This was followed by the L-PC, G-K, CAC, and G-Na systems, which exhibited 16.0%, 14.1%, 10.8%, and 10.4% higher residual strength values, respectively.

When the mechanical performance of the plaster mortars is evaluated together with the residual strength of the underlying concrete, it is observed that plasters with higher compressive strength do not always provide superior protective performance. The experimental results demonstrate that, although the CAC group exhibited high early-age strength and the highest absolute compressive strength values, the systems providing the most effective protection for the underlying concrete at elevated temperatures varied depending on the exposure temperature. Geopolymer-based plasters, particularly the G-K system, exhibited higher protective effectiveness in the 100–300 °C range, whereas Portland cement-based systems demonstrated more stable protective behavior, especially in the 500–700 °C range. These findings indicate that the high-temperature performance of the protected concrete is influenced not only by the mechanical properties of the plaster mortar but also by the interaction between the plaster layer and the underlying concrete during thermal exposure. Overall, the results demonstrate that plaster systems effectively preserve the residual strength of the underlying concrete after thermal exposure, and that their protective performance strongly depends on both binder type and exposure temperature.

### 3.2. UPV

**Figure 4:**
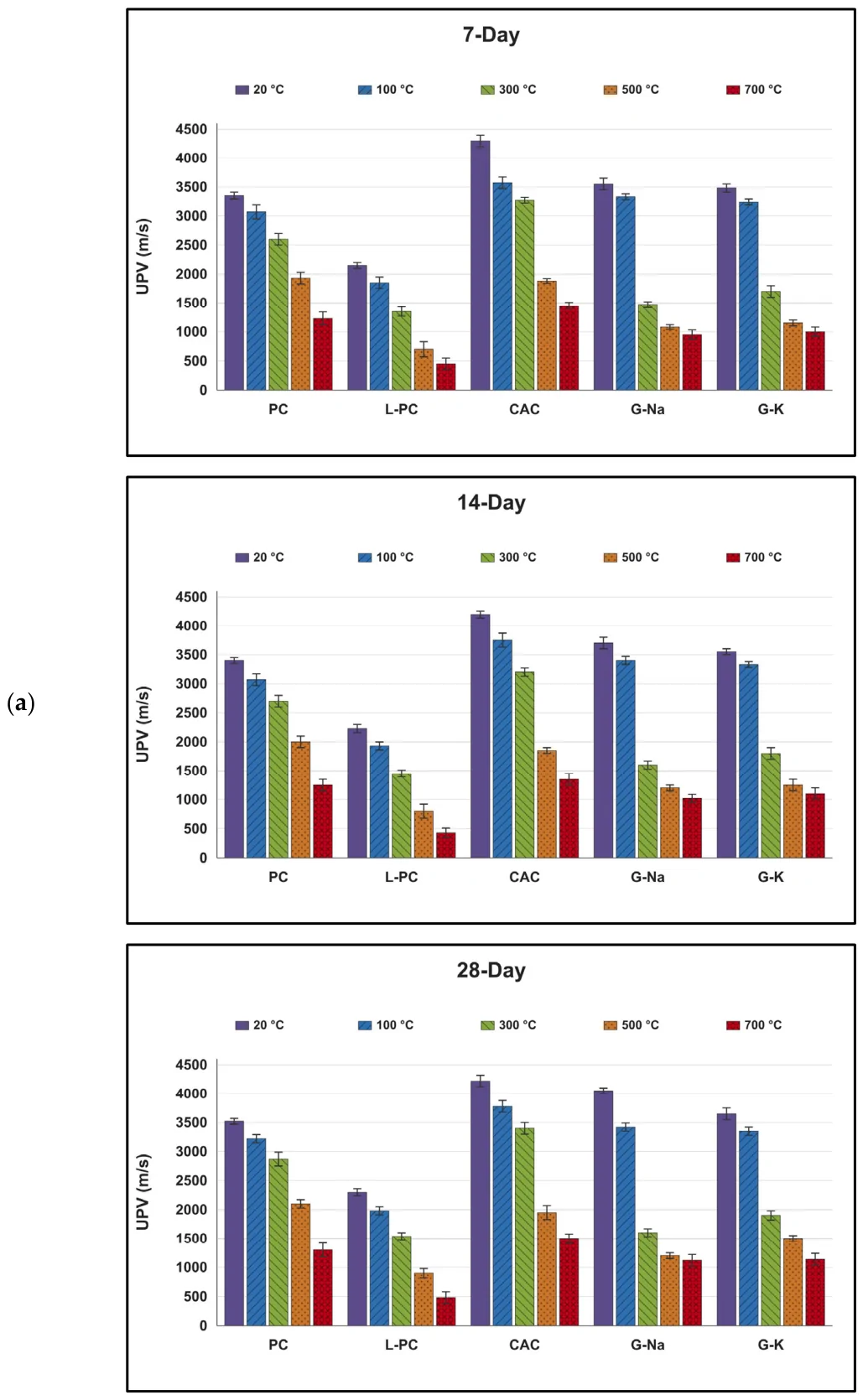

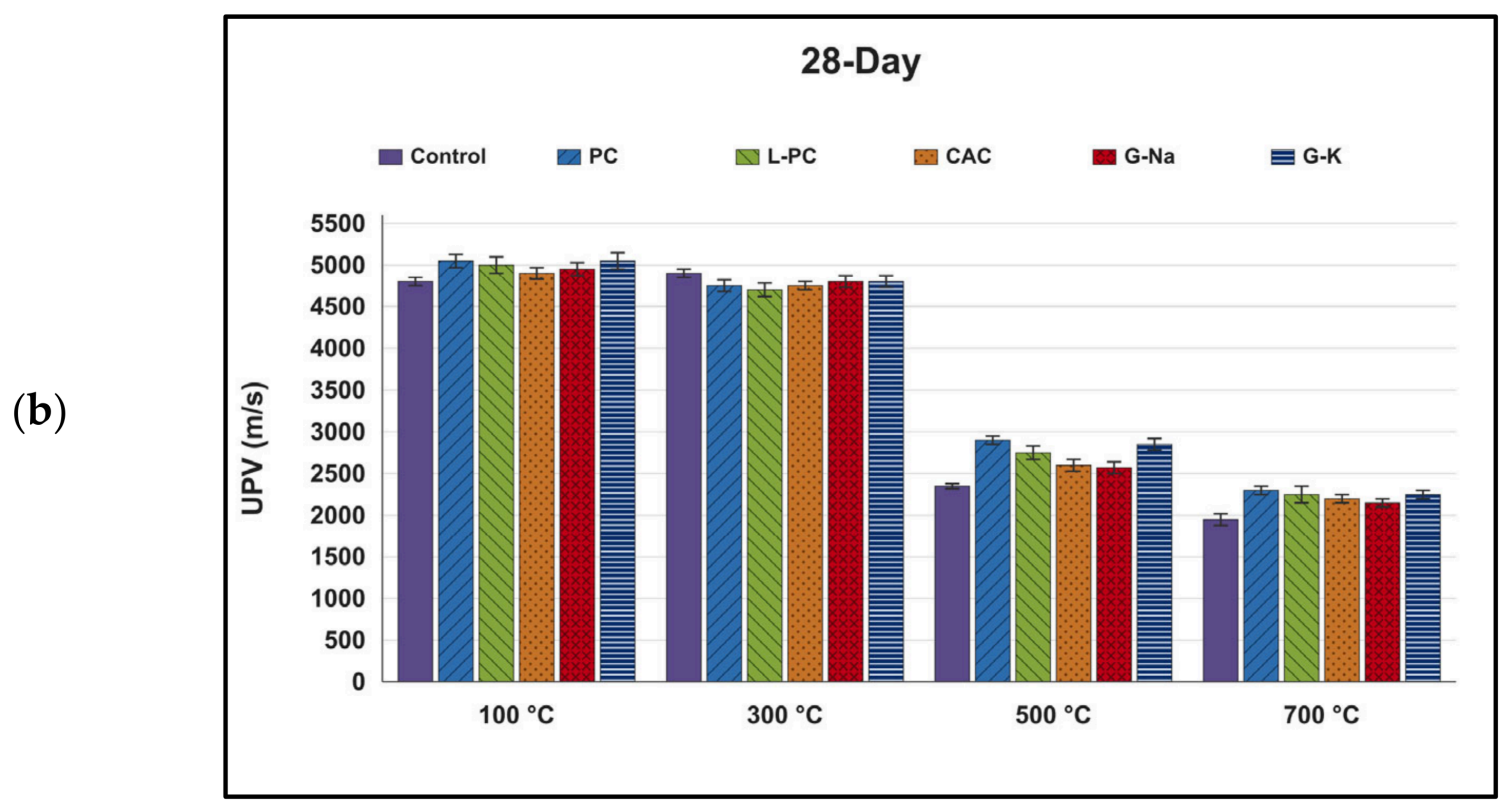
UPV values of the samples: (**a**) plaster mortar samples at 7, 14 and 28 days; (**b**) interior concrete samples at 28 days.

The UPV values of plaster mortar and plastered concrete samples with different binder compositions after high-temperature exposure are given in Figure 4. The results obtained demonstrate the effect of the structural damage occurring within the specimens as a result of the temperature increase on their UPV values. As shown in Figure 4a, the ultrasonic pulse velocity (UPV) results of the plaster mortars produced with different binder types indicate that both curing time and elevated temperature significantly affected the microstructural integrity of the materials. In general, an increase in UPV values was observed with increasing curing time across all binder systems, in agreement with previous studies reported in the literature [73,74]. In this situation, the hydration reactions that progress as the curing time increases cause the matrix structure to become denser and porosity to decrease, resulting in higher UPV values [75,76]. Under the reference condition of 20 °C, the CAC group exhibited the highest UPV values, whereas the L-PC group showed the lowest UPV values. Under the influence of high temperatures, UPV values decreased for all binders, and this decrease became more pronounced particularly after 300 °C. Water evaporation, which begins around 100 °C, causes a slight decrease in UPV values due to water’s higher sound wave velocity compared to air; this decrease continues up to 300 °C as a result of the removal of free and physically bound water and the onset of microcrack formation [77,78]. The marked acceleration in the decrease in sound wave velocity at temperatures above 300 °C is attributed to microstructural degradation caused by increased porosity and the resulting crack formation [79,80]. At temperatures up to 500 °C, voids formed by the evaporation of free and adsorbed water in concrete negatively affect ultrasonic wave transmission, leading to a decrease in UPV values [81]. Significant UPV losses occurred in all systems at 500 °C and 700 °C. These losses indicate that thermal damage caused by high temperatures disrupts the material’s internal structure, leading to porosity, void formation, and crack propagation [80,82,83,84]. Considering the retention of UPV after exposure to 700 °C, the PC system exhibited the highest resistance with a UPV reduction of 62.3%, followed by CAC (64.4%), G-K (68.0%), G-Na (71.8%), and L-PC (78.8%). These findings indicate that the extent of microstructural damage induced by high temperatures strongly depends on the binder type.

The 28-day ultrasonic pulse velocity (UPV) results of the interior concrete specimens obtained from plastered concrete specimens and the unplastered control concrete specimens are presented in Figure 4b. These results clearly demonstrated the protective effect of plaster systems on the integrity of the concrete matrix. At temperatures of 100 °C and 300 °C, the UPV values of plastered systems remained close to those of the control concrete, indicating that the internal structure was largely preserved at low and moderate temperatures. At temperatures of 500 °C and 700 °C, a significant decrease in UPV was observed in all systems; however, the plastered specimens maintained higher values compared to the control concrete. At 500 °C, the PC system exhibited the highest protective effect, providing a 22.2% higher UPV value than the unplastered control, followed by G-K (20.4%), L-PC (17.1%), CAC (10.7%), and G-Na (9.5%). Similarly, at 700 °C, the PC system showed the highest preservation of internal structural integrity, with an 18.3% higher UPV value compared with the control concrete, followed by L-PC (15.9%), G-K (14.6%), CAC (12.8%), and G-Na (9.5%). These results indicate that the plaster layer provides protection to the internal structure of the concrete and limits the development of internal damage during high-temperature exposure, as reflected by the higher UPV values of the plastered systems compared with the unplastered control concrete.

When the UPV performance of plaster mortars was evaluated together with the ultrasonic response of the concrete matrix, it was observed that plasters exhibiting higher initial UPV values did not necessarily provide the highest protective performance. In particular, although the CAC group exhibited high UPV values under reference conditions, which can be attributed to its dense microstructural characteristics and the nature of its hydration products, it showed more pronounced structural degradation at elevated temperatures. In contrast, PC and geopolymer systems exhibited a more balanced preservation of internal structural integrity across different temperature ranges. Overall, the results demonstrate that the reduction in UPV values is consistent with the loss of compressive strength and confirm that plaster systems effectively delay the development of internal damage in the concrete matrix during high-temperature exposure.

### 3.3. The Relationship Between the Compressive Strength of the Specimens and UPV

**Figure 5:**
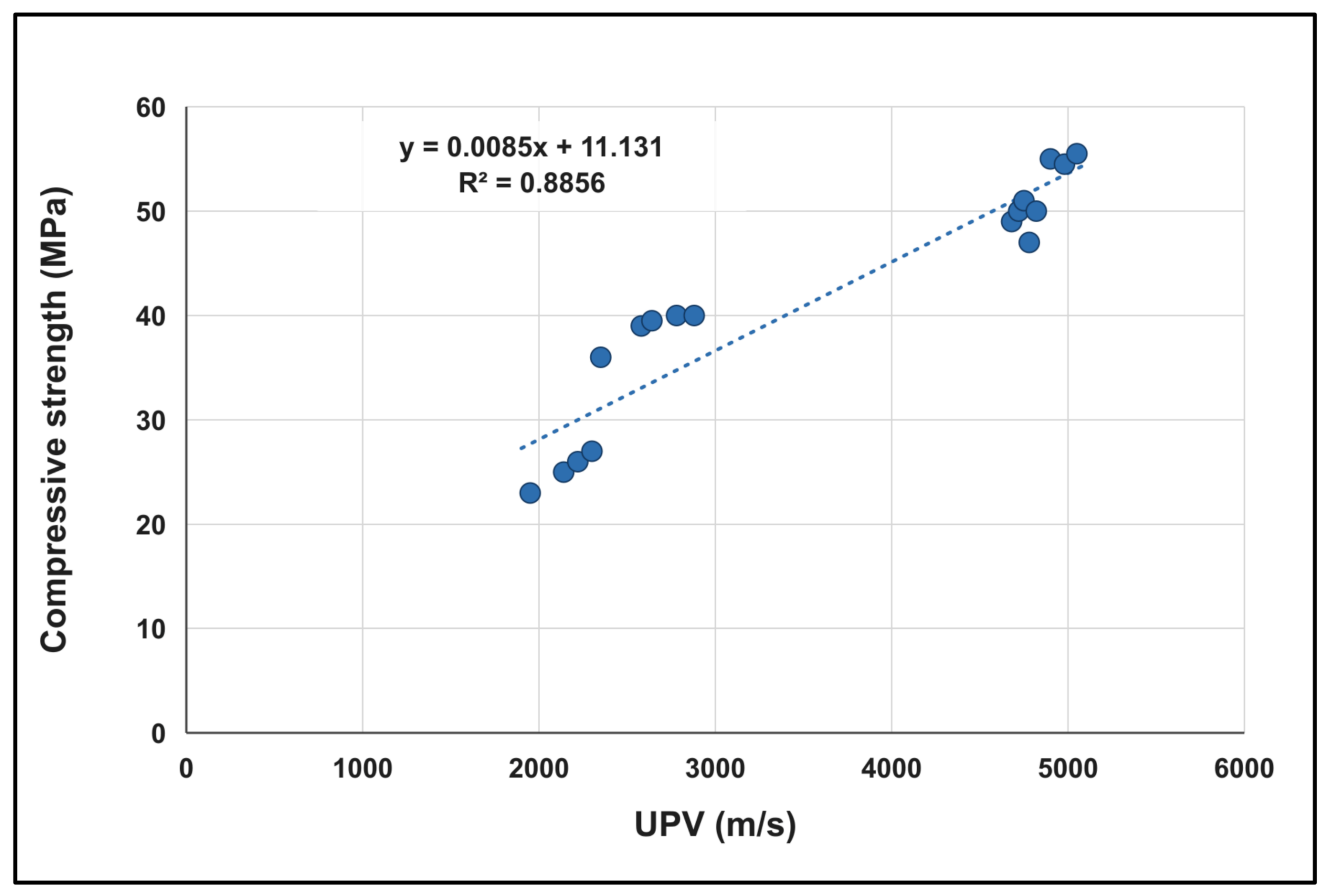
The relationship between compressive strength and UPV.

Figure 5 shows the relationship between the 28-day compressive strength and ultrasonic pulse velocity (UPV) values of the interior concrete specimens obtained from plastered concrete specimens exposed to elevated temperatures. A strong linear relationship was observed between the compressive strength and UPV values of the interior concrete specimens. Regression analysis yielded a high coefficient of determination (R^2^ ≈ 0.89), indicating that UPV effectively reflects the residual mechanical performance of concrete after high-temperature exposure [83,85]. This relationship suggests that internal damage caused by high temperatures, including microcrack formation and pore structure degradation, is reflected in the ultrasonic wave propagation characteristics. Moreover, surface observations do not necessarily reflect the internal condition of cementitious materials, and surface responses should therefore be interpreted together with complementary indicators of internal condition and transport-related performance [86]. Therefore, within the materials and temperature range investigated in this study, UPV can be considered a reliable non-destructive indicator for assessing the mechanical integrity of concrete after high-temperature exposure [87,88].

### 3.4. Water Absorption Ratios of the Specimens After Exposure to Elevated Temperatures

**Figure 6:**
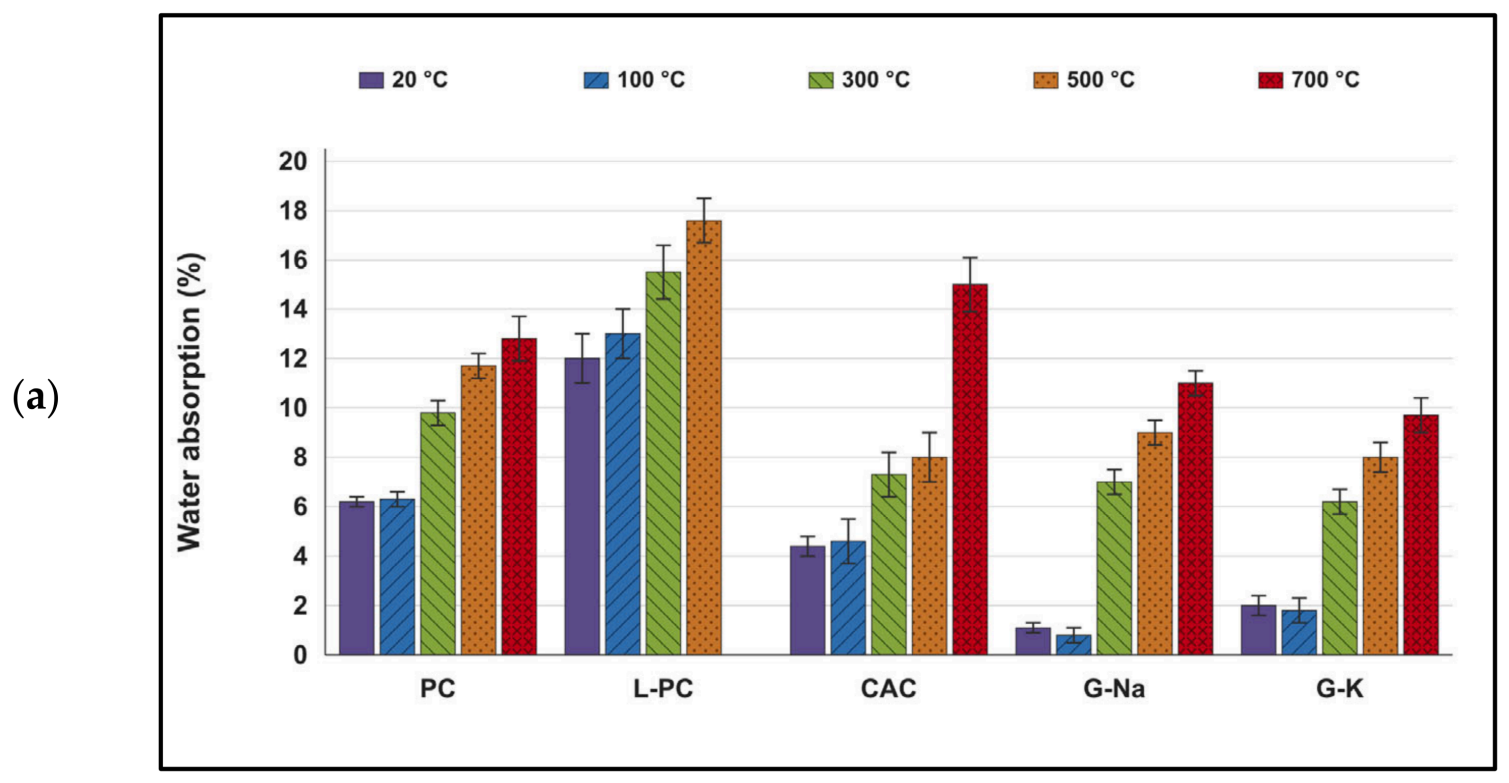

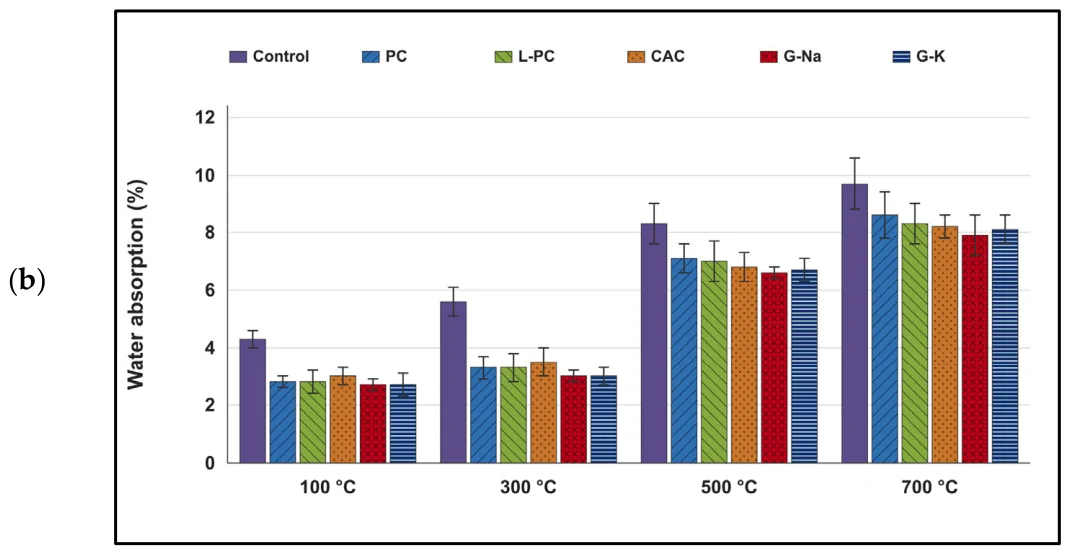
28-day water absorption rates: (**a**) plaster mortar samples, (**b**) interior concrete samples.

The water absorption rates of plaster mortar and plastered interior concrete specimens with different binder compositions after exposure to high temperatures are shown in Figure 6. The results obtained demonstrate the effect of changes in the pore structure and microstructure of the specimens, which occur with increasing temperature, on their water absorption rates.

For the plaster mortars, as presented in Figure 6a, the water absorption test results obtained for different binder systems revealed that high temperatures significantly affected the pore structure of the materials [89]. In general, as the exposure temperature increased, the amount of voids within the sample increased, resulting in a rise in concrete porosity and water absorption rates [90,91,92,93]. The water absorption values of PC, L-PC, CAC, G-Na, and G-K systems increased from 6.20%, 12.06%, 4.35%, 1.03%, and 1.92% at 20 °C to 9.79%, 15.57%, 7.32%, 6.93%, and 6.22% at 300 °C, respectively. Although the water absorption values of all binder systems remained relatively stable at 100 °C, a significant increase was observed at temperatures of 300 °C and above, consistent with previous studies reported in the literature [94]. This increase, observed following exposure to a temperature of 300 °C, is related to increased water ingress through microcracks formed by the high temperature and the resulting permeability channels [89]. In the PC and L-PC groups, water absorption values increased gradually with rising temperature, and the L-PC group exhibited the highest water absorption values at all temperature levels. While the CAC group showed a moderate increase, geopolymer systems (particularly G-Na) exhibited very low water absorption values at low temperatures; however, these values increased rapidly at temperatures of 300 °C and above. The increase in cracks and internal structural damage on the concrete surface following heating up to 500 °C and 700 °C caused a significant rise in the specimens water absorption capacity [95,96]. At 700 °C, the G-K system exhibited the lowest water absorption value (9.66%), followed by G-Na (11.09%), PC (12.85%), and CAC (14.94%) systems. It should be noted that the water absorption value of the L-PC system at 700 °C could not be determined because the specimen was severely damaged and fragmented under high-temperature exposure. These results indicate that the G-K system provided the highest resistance against pore structure deterioration among the investigated binder systems.

The water absorption results of the interior concrete specimens obtained after removing the plaster layer from plastered concrete specimens and the unplastered control concrete specimens after high-temperature exposure are presented in Figure 6b. The fact that the water absorption of the plaster-coated samples is lower than that of the uncoated samples shows that plaster systems provide a protective effect by reducing the permeability in the internal structure of the concrete [97]. At all temperature levels, plastered systems exhibited lower water absorption values compared to the control concrete, indicating that the plaster layer limited the development of heat-induced microcracks and the formation of open pores. A significant improvement was observed among the plastered systems, particularly at 100 °C and 300 °C, with geopolymer-based plasters demonstrating superior performance through lower water absorption values. At 300 °C, the G-Na system exhibited the lowest water absorption value (2.96%), followed by G-K (3.05%), L-PC (3.30%), PC (3.32%), and CAC (3.52%), whereas the control concrete showed a higher value of 5.62%. At 500 °C and 700 °C, water absorption values increased in all systems; however, the plastered specimens continued to exhibit lower permeability compared to the control concrete. At 700 °C, the G-Na system exhibited the lowest water absorption value (7.78%), representing a 19.5% reduction compared with the unplastered control concrete (9.66%), followed by CAC (8.14%), G-K (8.13%), L-PC (8.30%), and PC (8.63%). These results indicate that the plaster systems reduce the internal temperature by mitigating the thermal effect on the concrete matrix, thereby limiting the formation of cracks and capillary voids and preserving the structural integrity [97].

As the temperature increased, an increase in water absorption was observed in all systems, and the magnitude of this increase varied depending on the binder type. In general, geopolymer-based systems exhibited lower water absorption values in plaster mortars and provided a significant barrier effect for protecting the interior concrete under high-temperature exposure. The increase in water absorption was found to be consistent with the decrease in UPV values and the loss of compressive strength, demonstrating that plaster systems limited the development of internal damage by reducing the permeability of the concrete matrix. These findings indicate that high-temperature performance is governed not only by mechanical strength but also by pore structure characteristics and capillary water transport behavior.

### 3.5. Mass Loss Ratios of the Specimens After Exposure to Elevated Temperatures

**Figure 7:**
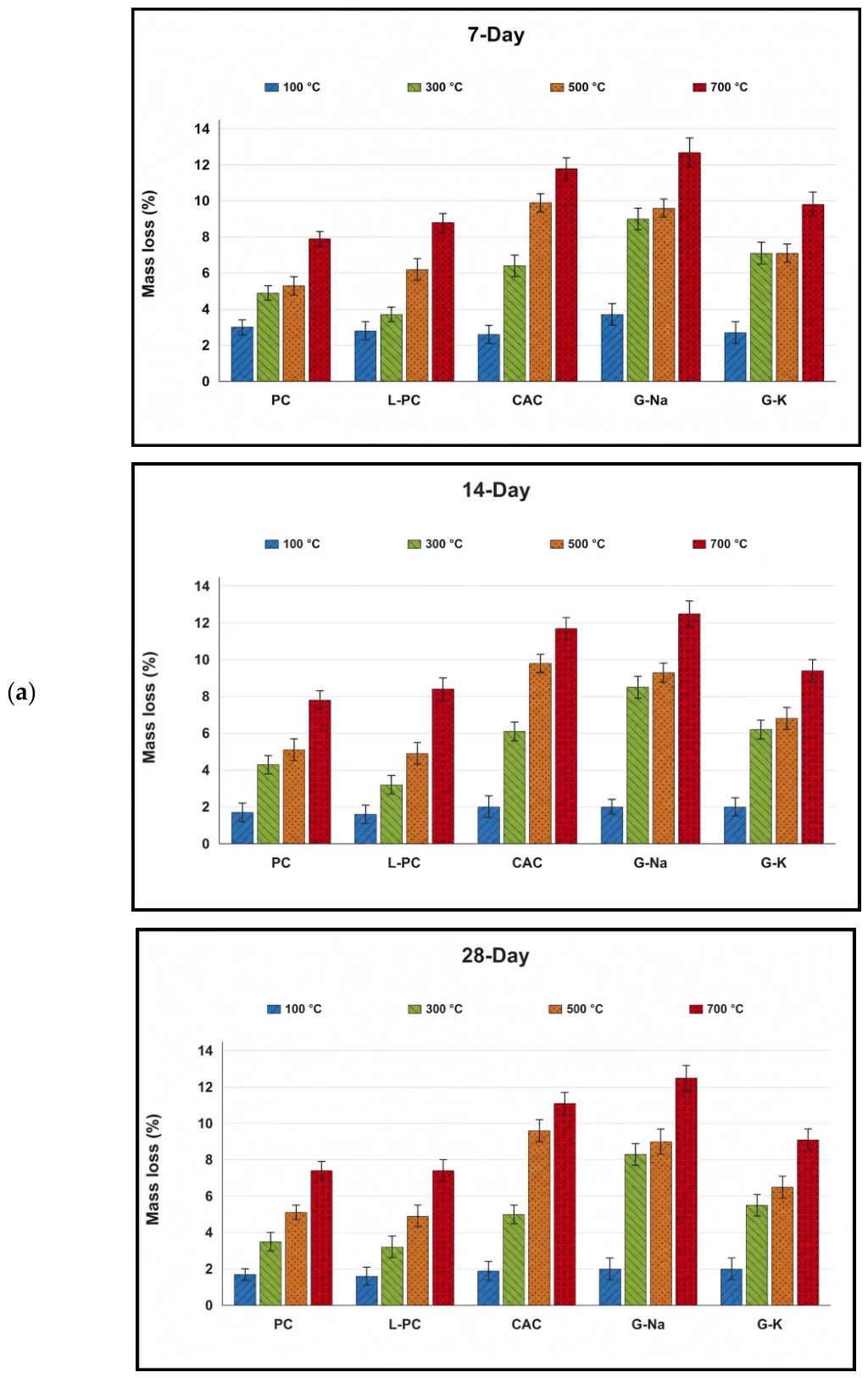

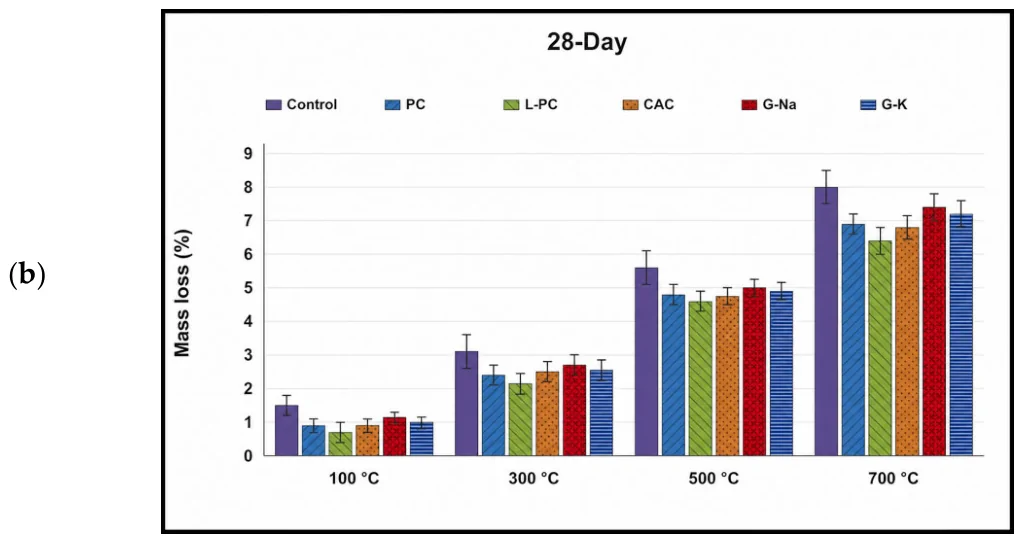
Mass loss ratios: (**a**) plaster mortar specimens cured for 7, 14, and 28 days; (**b**) 28-day concrete specimens.

The mass loss ratios of plaster mortar and plastered interior concrete samples with different binder compositions after high-temperature exposure are given in Figure 7. The results obtained demonstrate the effect of moisture loss in the samples with increasing temperature and the physical and chemical changes in the binder components on the mass loss behavior.

For plaster mortars, as presented in Figure 7a, the mass loss results obtained from different binder systems showed that increasing temperature affected the thermal stability of the materials. In all binder systems, mass loss increased with rising temperature, and this increase became particularly pronounced after 300 °C; this finding is consistent with results reported in the literature [98,99]. The initial mass loss that occurs in the low-temperature range (approximately 100–200 °C) is due to the evaporation of free water present on the surface and within the pores of the samples [98,100,101,102]. The increase in mass loss following exposure to 300 °C is related to the decomposition of hydration products, the removal of free and bound water, and the thermal decomposition of the binder phases. At 300 °C, the L-PC system exhibited the lowest mass loss value (3.23%), followed by PC (3.53%), CAC (5.00%), G-K (5.48%), and G-Na (8.28%). With increasing temperature, the differences between the binder systems became more evident. At 700 °C, the PC system showed the lowest mass loss value (7.37%), followed by L-PC (7.41%), G-K (9.12%), CAC (11.15%), and G-Na (12.46%). While the CAC and geopolymer (G-Na and G-K) systems exhibited higher mass loss, particularly at 500 °C and 700 °C, the PC and L-PC groups demonstrated lower and more controlled mass loss behavior. The mass loss observed in the PC and L-PC groups after high-temperature exposure can be partly attributed to the decomposition of calcium hydroxide (CH) [98,102]. According to the literature, mass loss in geopolymer samples shows a rapid increase in the low-temperature range (approximately 100–200 °C), while in the medium-temperature range (200–400 °C) this rate of increase decreases, and at higher temperatures (above 400 °C) a pronounced mass loss occurs once again [103]. These results demonstrate that binder type is a determining factor in the thermal degradation behavior of plaster mortars.

As shown in Figure 7b, the mass loss results obtained from the plastered interior concrete and unplastered control concrete specimens demonstrated that plaster systems provided a protective effect against thermal degradation of the concrete matrix. At all temperature levels, the plastered specimens exhibited lower mass loss values compared to the control concrete, suggesting that the plaster layer contributed to protecting the interior concrete from thermal degradation and associated moisture loss. At 100 °C and 300 °C, the protective effect of plaster systems was more pronounced, with the L-PC system exhibiting the lowest mass loss values (0.63% and 2.21%, respectively), followed by PC (0.87% and 2.45%) at 100 °C and 300 °C, respectively. With increasing temperature, the differences between the systems became more evident. At 700 °C, the L-PC system showed the lowest mass loss value (6.38%), followed by CAC (6.83%), PC (6.88%), G-K (7.17%), and G-Na (7.43%), whereas the control concrete exhibited the highest value (8.02%). These results indicate that the plaster layer effectively restricted thermal degradation and mass loss of the interior concrete by reducing the severity of heat-induced damage. Although geopolymer-based plaster systems showed relatively higher mass loss values at elevated temperatures compared with PC-based systems, they still provided a protective effect compared with the unplastered control concrete.

When the mass loss behavior of plaster mortars was evaluated together with the thermal mass loss of the interior concrete, it was observed that the mass stability of plaster layers did not always correspond to the lowest degradation level of the interior concrete. CAC- and geopolymer-based systems exhibited higher mass loss values at elevated temperatures, whereas PC- and L-PC-based systems demonstrated more stable thermal behavior. Overall, the results indicate that plaster systems effectively reduce the mass loss of the interior concrete, and that this protective effect varies depending on the temperature level and binder type. This indicates that high-temperature performance is governed not only by mechanical strength and permeability characteristics but also by the thermal degradation behavior reflected by mass loss.

### 3.6. Bulk Density of the Specimens After Exposure to Elevated Temperatures

**Figure 8:**
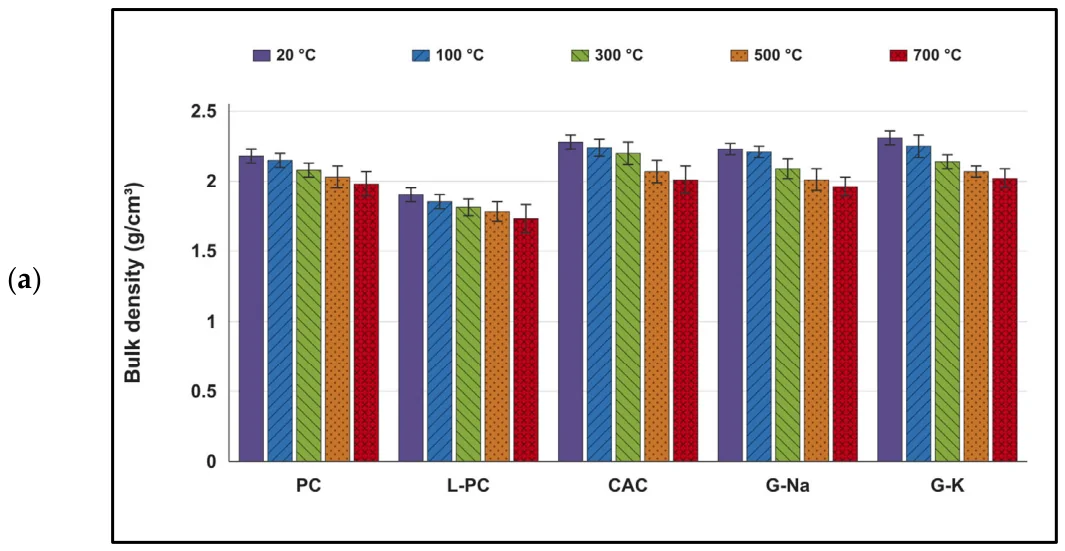

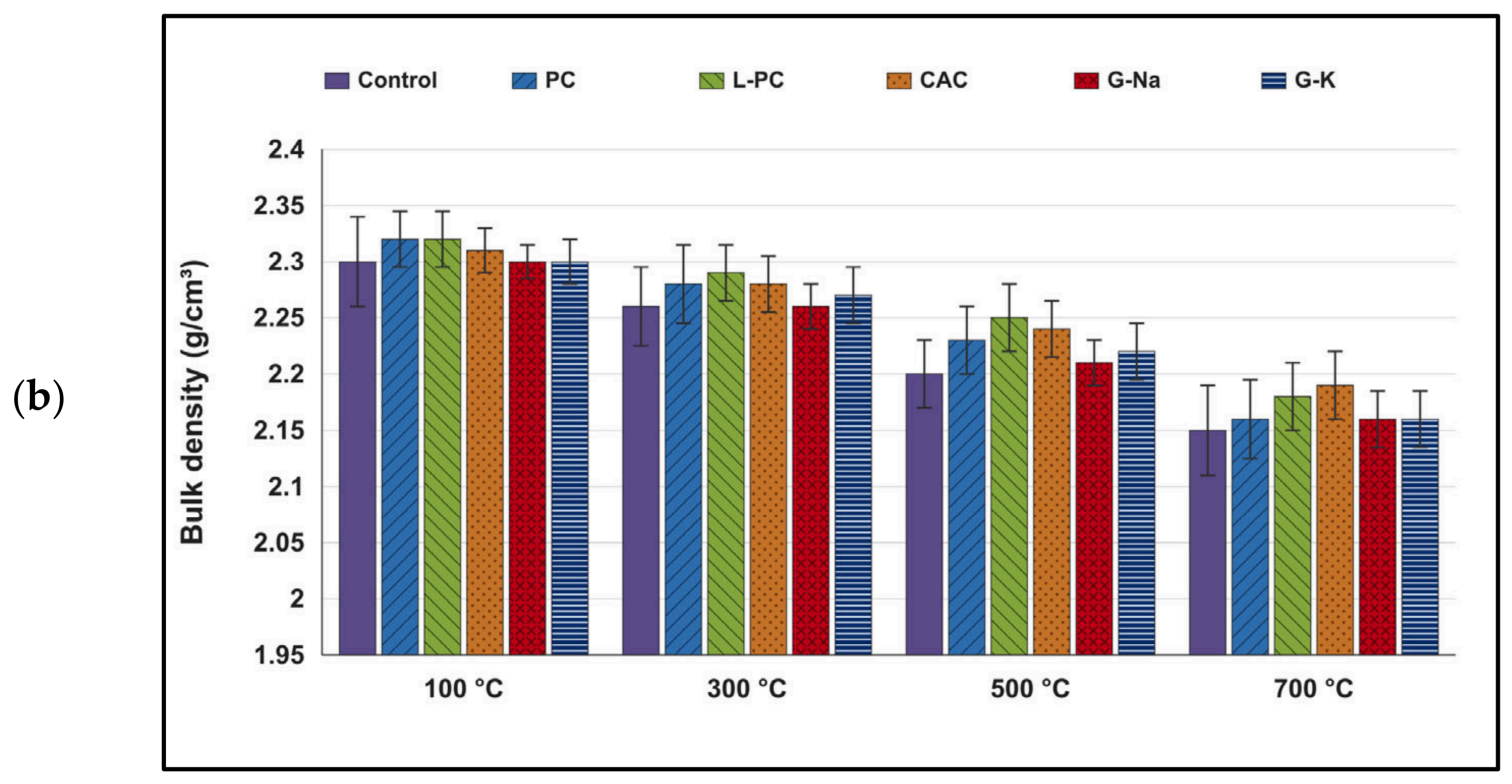
Bulk density values of 28-day samples: (**a**) plaster mortar samples; (**b**) interior concrete samples.

The bulk density values of plaster mortar and plastered interior concrete specimens with different binder compositions after exposure to high temperatures are shown in Figure 8. The results indicate that the changes in the density values of the specimens with increasing temperature are related to water loss and microstructural degradation.

For plaster mortars, as presented in Figure 8a, the bulk density results obtained from different binder systems showed that increasing temperature caused a gradual reduction in the density values of the materials. This behavior is associated with moisture loss, decomposition of hydration products, and microstructural degradation under elevated temperatures [104]. The decrease observed at low temperatures can be explained by the evaporation of water within the mortar [105]. As the exposure temperature increased, the progressive reduction in bulk density values in all binder systems indicated the formation of additional voids and deterioration of the internal structure. Among the investigated systems, CAC and G-K groups maintained relatively higher bulk density values throughout the entire temperature range, whereas the L-PC system exhibited the lowest density values at all temperatures. The percentage reduction in bulk density from 20 °C to 700 °C followed the order of PC (7.9%), L-PC (9.4%), G-Na (11.7%), CAC (11.8%), and G-K (12.2%). Although PC exhibited the lowest relative reduction in bulk density, CAC and G-K maintained comparatively higher bulk density levels after high-temperature exposure. These results indicate that the binder type plays a critical role in controlling pore development and microstructural degradation under high-temperature exposure.

As shown in Figure 8b, the bulk density results of the plastered and control interior concrete specimens indicate that the plaster systems provided a limited contribution to the preservation of density under high-temperature exposure. Although the plastered specimens exhibited slightly higher bulk density values compared with the control concrete, the differences remained relatively small at all temperature levels. The difference became more noticeable at elevated temperatures; however, the overall effect of the plaster layer on bulk density retention was limited. Among the investigated systems, CAC and L-PC exhibited relatively better density retention, while geopolymer-based systems showed a comparatively higher reduction in density.

When the bulk density behavior of plaster mortars was evaluated together with the change in bulk density of the interior concrete, it was observed that systems with higher initial density do not always result in better structural stability at elevated temperatures. In particular, although geopolymer-based plasters exhibited high initial performance, they experienced a more pronounced density reduction with increasing temperature. Overall, the results demonstrate that plaster systems contribute to limiting density loss in the interior concrete, and that this effect varies depending on temperature level and binder type. These findings indicate that high-temperature performance is governed not only by mechanical strength but also by structural density and the formation of internal voids.

### 3.7. Visual Appearance of the Specimens After Exposure to Different Temperatures

**Figure 9:**
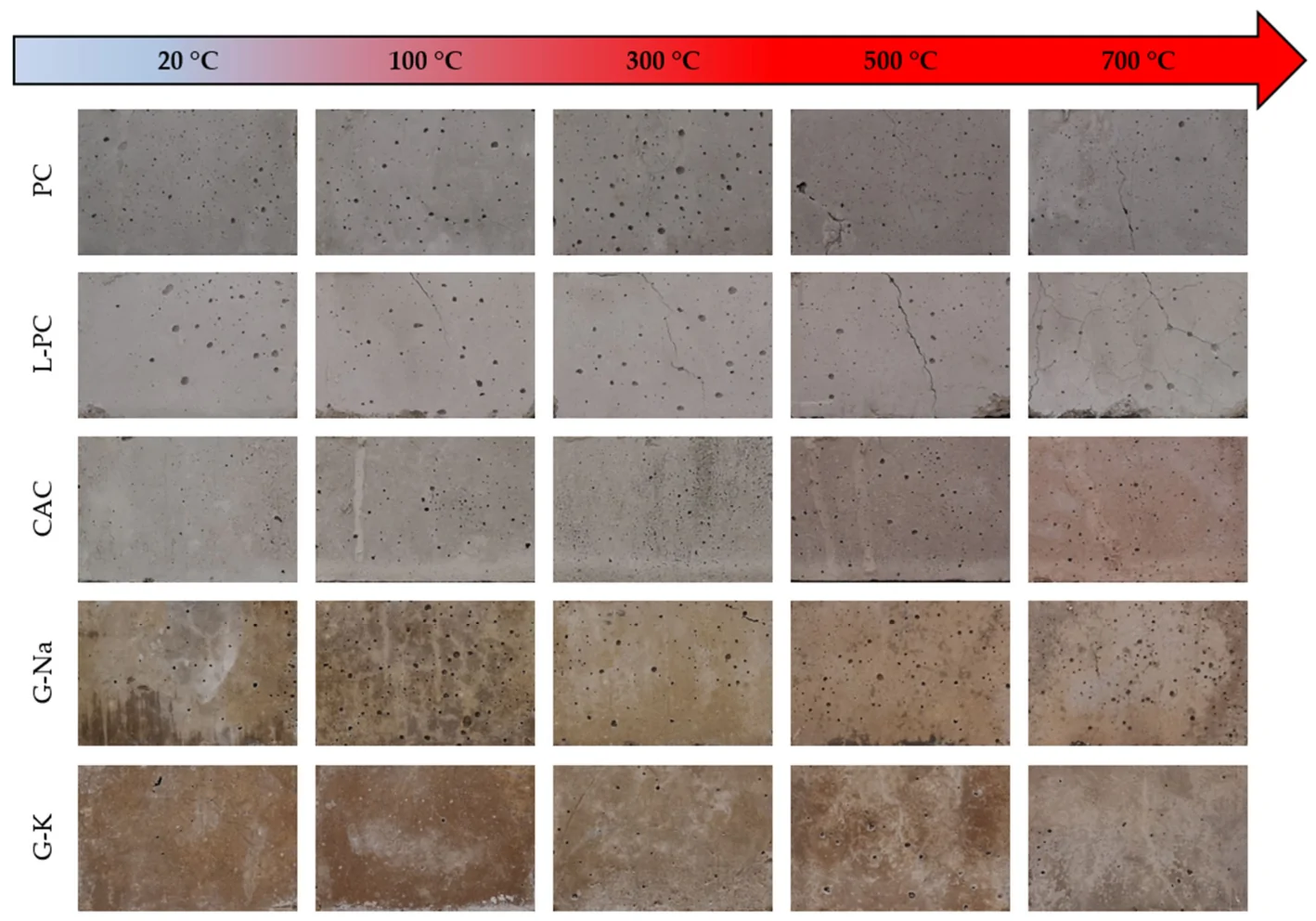
Photographs of the 28-day-old specimens after exposure to different temperatures.

Figure 9 shows the visual appearance of plastered concrete specimens after exposure to different temperatures. The observations indicate that physical changes and crack formation occurred on the specimen surfaces as the temperature increased.

The effects of high temperature on plastered specimens were evaluated through visual examinations of different groups (PC, L-PC, CAC, G-Na, and G-K). In general, the increase in temperature caused significant effects on crack formation, color changes, and spalling on the specimen surfaces. The investigation of these visual changes can be used as an important method for the preliminary assessment of post-fire damage [106,107,108,109,110]. Crack formation occurs as a result of water loss due to high temperatures, dehydration of binder gels, and microstructural deterioration [107,111]. While no significant deterioration was observed in the PC group specimens up to 500 °C, crack formation started after this temperature, and at 700 °C, the cracks both widened and increased in number [106]. The color change progressed from gray tones toward light gray [108,109]. In the L-PC group, crack formation started at 100 °C, and the crack density and width increased significantly with increasing temperature. At 700 °C, the coating integrity was severely deteriorated; however, no significant color change was observed. In the CAC group specimens, wide and dense cracks did not occur at high temperatures, whereas fine and limited cracks were detected. The color change was observed in gray tones at low temperatures, while it transformed into a gray color with orange tones with increasing high-temperature exposure. A similar color change behavior has been reported in the literature [112]. In the G-Na group, no significant crack development was observed except at 700 °C, where only fine and limited cracks were formed. In the G-K group, crack formation remained at a minimal level, and no significant damage was observed. In geopolymer specimens, a color change from the initial brown tones toward light gray colors can be observed due to the effect of high temperature [111]. The color change in the geopolymer group is considered to be caused by the oxidation of mineral components [106,110,113].

### 3.8. Microstructural Analysis

#### 3.8.1. SEM

**Figure 10:**
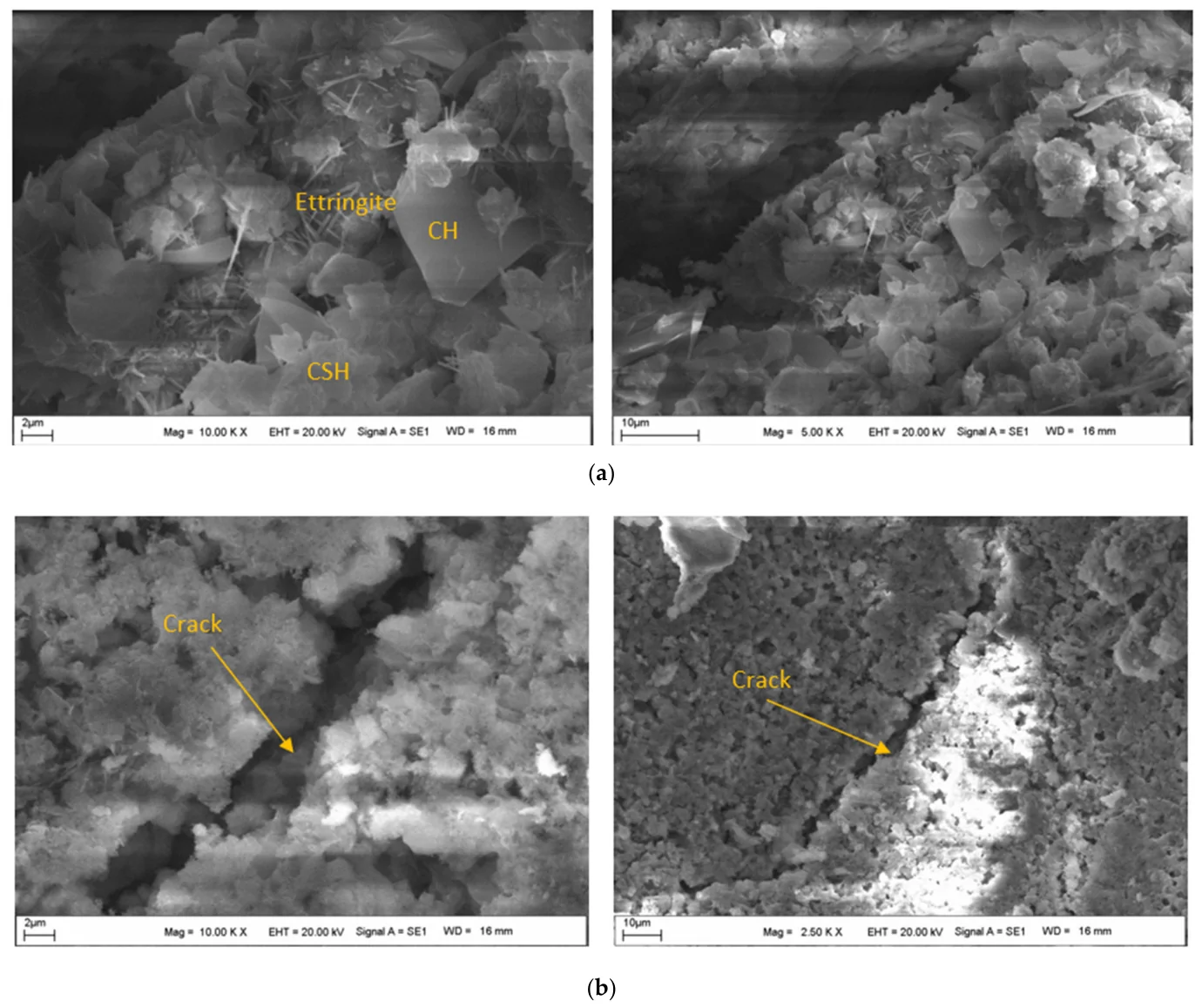

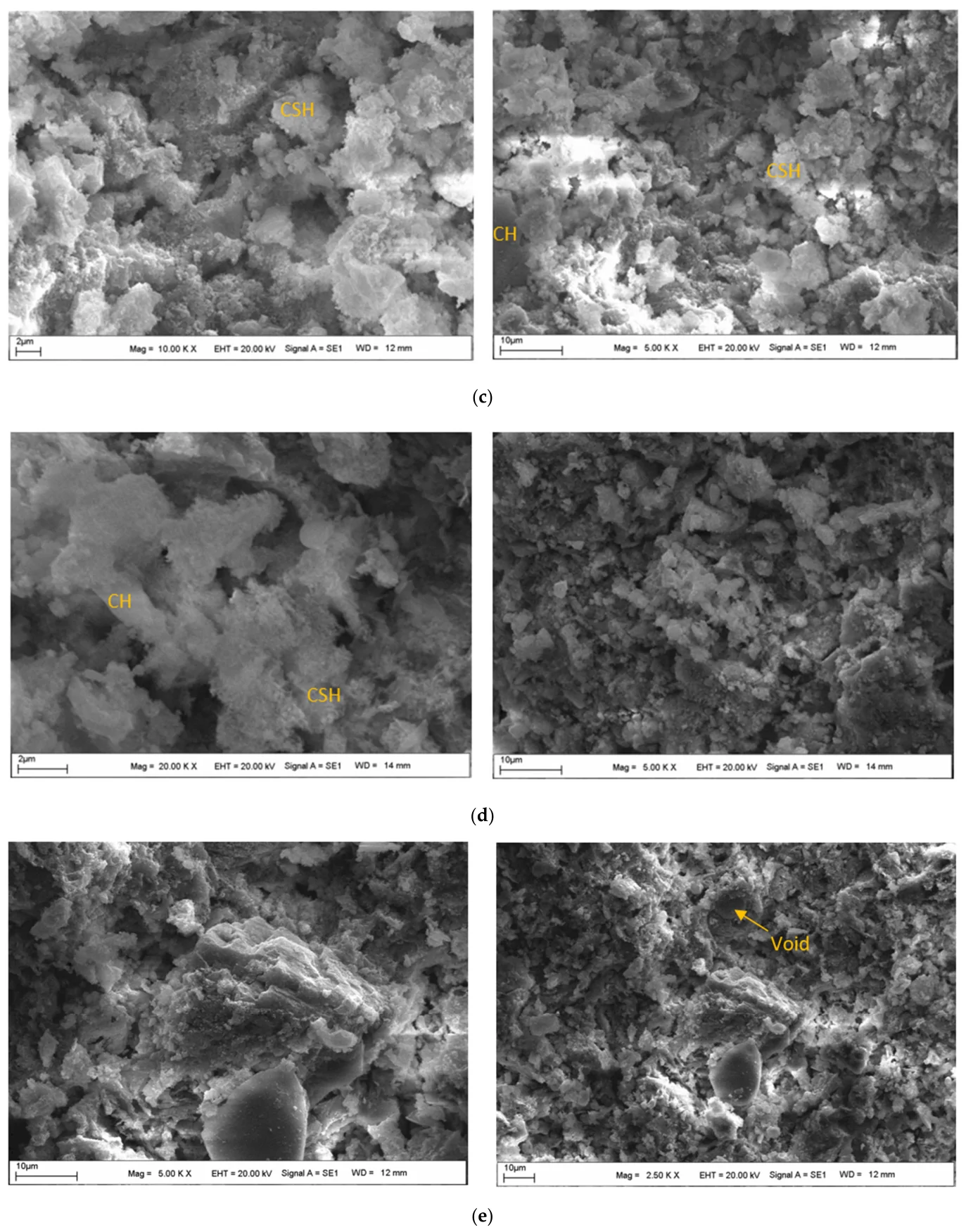

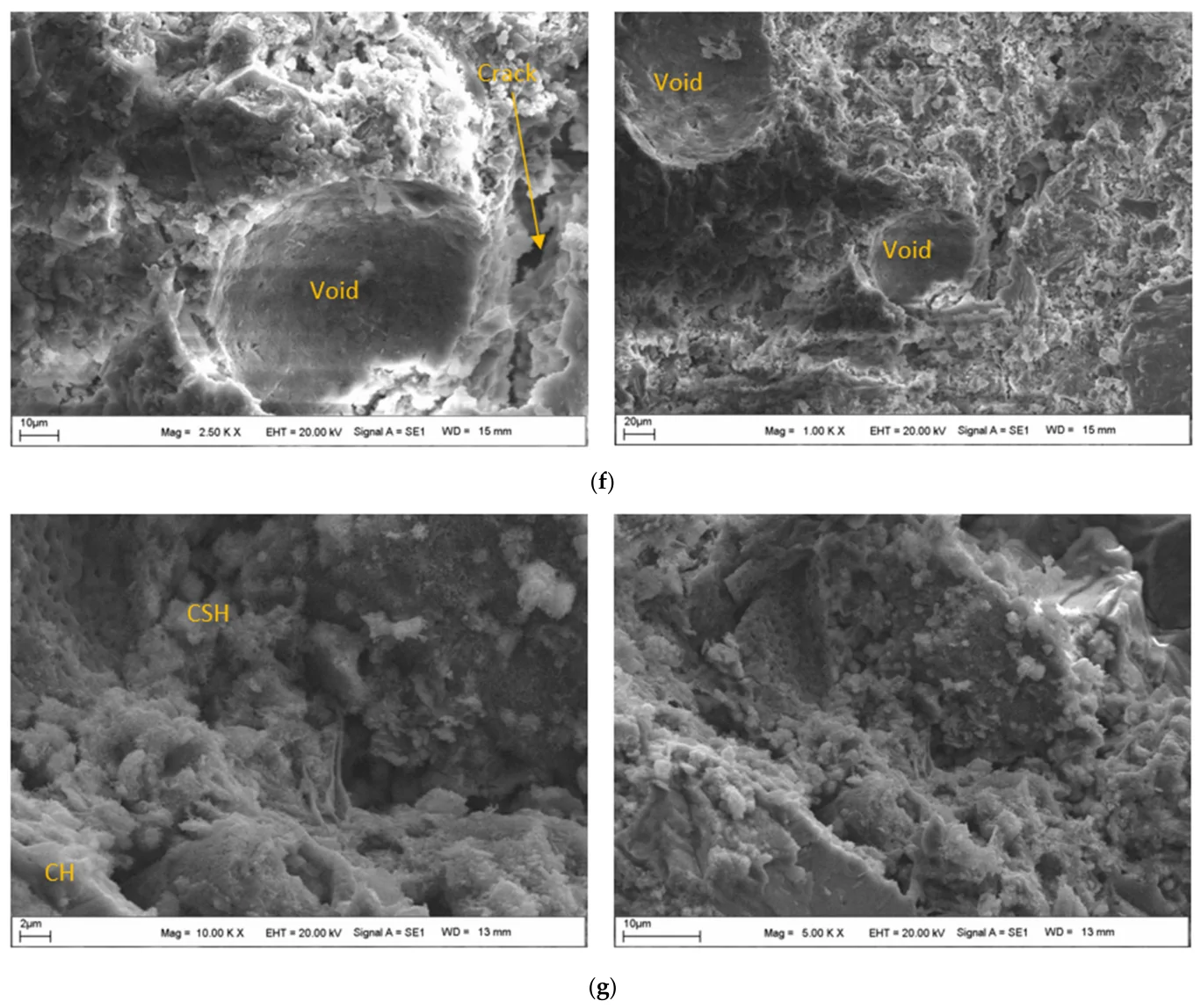
SEM images of 28-day-old specimens before and after high-temperature exposure: (**a**) unheated unplastered control concrete specimen; (**b**) unplastered control concrete specimen exposed to 700 °C; and (**c**–**g**) interior concrete obtained from PC, L-PC, CAC, G-Na, and G-K plaster-coated specimens, respectively, after exposure to 700 °C and removal of the plaster layer.

Samples were taken from the control concrete specimens and the interior concrete specimens before (room temperature) and after (700 °C) high-temperature exposure and examined using SEM. The obtained images are presented in Figure 10. For each specimen group, two SEM images are shown from the same sampling region at different magnifications.

In the SEM images of the interior concrete specimens with removed plaster and the control concrete specimens, C–S–H-, CH-, and ettringite-like morphologies, as well as pores and cracks, were observed. Microcracks were formed in the specimens exposed to a temperature of 700 °C. The changes in the microstructure of concrete are associated with microcrack formation, an increase in pore structure, partial deterioration of the C–S–H gel, and deformation of calcium hydroxide crystals [91,114]. These formed microcracks are considered to be responsible for the reduction observed in the compressive strength values of the specimens [115,116]. These cracks may have resulted from the volumetric expansion occurring after dehydration and the water vapor released after heating [115].

#### 3.8.2. XRD Analysis

**Figure 11:**
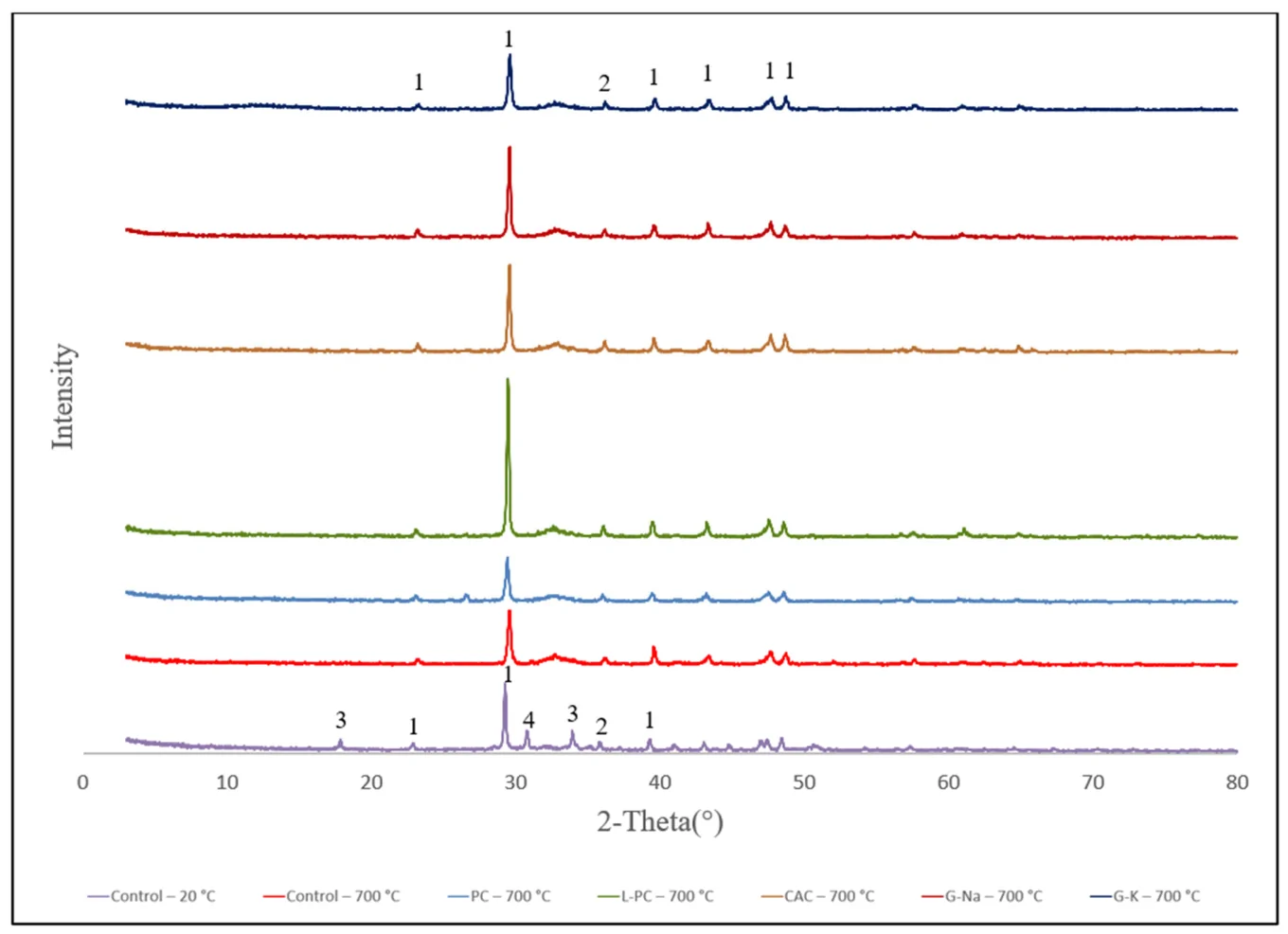
XRD patterns of 28-day-old specimens before and after high-temperature exposure: (1) calcite (CaCO_3_), (2) calcium silicate phases, (3) portlandite (Ca(OH)_2_), and (4) calcium silicate hydrate (C–S–H).

Samples were taken from the control concrete specimens and interior concrete specimens before and after high-temperature exposure (700 °C), and XRD analyses were performed. The graphs obtained as a result of the analyses are presented in Figure 11.

As a result of the analysis, calcite (CaCO_3_), portlandite (Ca(OH)_2_), calcium silicate phases, and calcium silicate hydrate were detected in the control and interior concrete specimens kept under laboratory conditions, whereas calcite (CaCO_3_) and calcium silicate phases were identified in the plaster-removed interior concrete specimens exposed to 700 °C. In the literature, X-ray analyses of unheated concrete (20 °C) indicate that calcite (C), portlandite (P), and hydrated calcium silicates (C–S–H) are the main crystalline phases [117,118]. The characteristic feature associated with C–S–H was no longer clearly distinguishable in the specimens after high-temperature exposure. This observation may be attributed to the dehydration and/or structural transformation of the C–S–H gel [103]. The almost complete disappearance of the portlandite peak at high temperatures is consistent with the results reported in the literature [118,119]. The disappearance of the portlandite peak and the loss of the characteristic C–S–H feature are consistent with the microstructural deterioration observed in the SEM images, including increased pore formation and microcracking. These changes in the hydrated cement phases and microstructure are also consistent with the reduction in compressive strength observed after exposure to 700 °C. The observed similarity between the XRD patterns of the unplastered control specimens and the interior concrete specimens obtained from the plaster-coated specimens after exposure to 700 °C is consistent with the literature reporting that the characteristic peaks of the cement matrix can be largely preserved at elevated temperatures [120].

## 4. Conclusions

In this study, the performance of plaster mortars produced using different types of binders, as well as the changes in compressive strength, ultrasonic pulse velocity (UPV), water absorption rate, mass loss rate, and bulk density of mortar and plastered concrete specimens after high-temperature exposure, was investigated. In addition, visual examinations and microstructural analyses using SEM and XRD were conducted to evaluate the damage mechanisms and changes in the internal structure of the specimens. The results obtained from the experimental investigations are summarized below.

- The mechanical and physical properties of plaster mortars produced with different binder types were significantly affected by curing time and high-temperature exposure. In general, increasing the curing time resulted in higher compressive strength and UPV values, while exposure to elevated temperatures, particularly at 300 °C and above, caused decreases in UPV, compressive strength, and bulk density, together with increases in water absorption and mass loss.
- The response of the plaster mortars to elevated temperatures varied depending on the binder type. Although the CAC group exhibited high early-age strength, it experienced significant strength losses under thermal exposure. The geopolymer systems (G-Na and G-K) showed relatively good performance at lower temperatures but experienced structural deterioration at higher temperatures. Visual examinations also showed that temperature increase caused crack formation and color changes in the plaster systems, with more pronounced surface deterioration observed in the PC and particularly L-PC groups, whereas the G-K and G-Na groups exhibited relatively limited surface deterioration.
- The application of plaster mortar provided a significant protective effect to the interior concrete during high-temperature exposure. Compared with the control concrete specimens, the plastered concrete specimens generally exhibited higher compressive strength and UPV values, as well as lower water absorption and mass loss. The bulk density results also indicated that the interior concrete structure was better preserved in the plastered specimens, demonstrating the protective contribution of the plaster layer against thermal damage.
- A strong correlation (R^2^ ≈ 0.89) was obtained between UPV and compressive strength in the interior concrete specimens exposed to different temperatures. This finding indicates that UPV can be used as an indicative parameter for assessing the residual mechanical performance of the investigated concrete specimens within the temperature range and material conditions considered in this study.
- SEM observations revealed hydration-product-like morphologies, together with pore and crack structures, in the control and interior concrete specimens. High-temperature exposure, particularly at 700 °C, resulted in increased microcrack formation and microstructural deterioration, which were associated with the reduction in compressive strength. XRD analyses identified portlandite, calcium silicate phases, and C–S–H in the control and interior concrete specimens, while changes in these phases were observed after exposure to 700 °C. These microstructural findings support the mechanical deterioration observed after high-temperature exposure.

Overall, the results indicate that plaster systems can improve the high-temperature performance of concrete by limiting the deterioration of its mechanical and physical properties and helping to preserve the integrity of the interior concrete. The protective effect of the plaster layer was reflected in higher residual strength, lower water absorption and mass loss, and better retention of bulk density after thermal exposure.

## Figures and Tables

**Table 1:** Chemical compositions and physical properties of binder materials.

Chemical Characteristics (%)	CEM I	CEM II/A-M (P-LL)	SLL	CAC	GGBFS
SiO_2_	18.76	19.50	1.75	6.20	32.47
Al_2_O_3_	5.19	6.01	0.35	50.00	9.94
Fe_2_O_3_	3.68	3.88	0.20	2.70	1.25
CaO	63.47	59.98	89.50	38.80	32.45
MgO	1.67	1.88	1.00	0.80	9.31
Cl_2_	0.01	0.01	-	0.01	0.01
**Physical Characteristics**					
Specific Weight (g/cm^3^)	3.12	3.06	-	3.25	2.86
Specific Surface Area (cm^2^/g)	3938	4266	-	4000	3996

**Table 2:** Mixing ratios of plaster mortars.

Materials/Parameters	PC	L-PC	CAC	G-Na	G-K
Cement, (g)	500	250	-	-	-
Lime, (g)	-	250	-	-	-
CAC, (g)	-	-	500	-	-
GGBFS, (g)	-	-	-	500	500
Sand, (g)	1500	1500	1500	1500	1500
Water, (g)	266.89	374.22	241.69	223.13	193.90
NaOH, (g)	-	-	-	89.25	-
KOH, (g)					108.78
Total liquid-to-binder ratio	0.53	0.75	0.48	0.62	0.60
Solution concentration (M)				10	10

**Table 3:** Nomenclature of the plaster mortars.

Composition of the Plaster Type	Nomenclature
Portland Composite Cement	PC
Lime + Portland Composite Cement	L-PC
Calcium Aluminate Cement	CAC
NaOH-activated GGBFS	G-Na
KOH-activated GGBFS	G-K

## Data Availability

The original contributions presented in this study are included in the article. Further inquiries can be directed to the corresponding author.

## References

[1] Sobuz M.H.R., Khan M.H., Islam M.R., Kabbo M.K.I., Alzlfawi A., Jameel M., Khan M.M.H. (2025). Combined Influence of Crushed Brick Powder and Recycled Concrete Aggregate on the Mechanical, Durability and Microstructural Properties of Eco-Concrete: An Experimental and Machine Learning-Based Evaluation. J. Mater. Res. Technol..

[2] Sowmya S., Pannem R.M.R. (2025). Integrating Bacteria and Fibres for Self-Healing Concrete: A Comprehensive Review of Synergistic Effects. Results Eng..

[3] Wang W., Yue Q. (2023). The Time Variation Law of Concrete Compressive Strength: A Review. Appl. Sci..

[4] Qu F., Li W., Dong W., Tam V.W.Y., Yu T. (2021). Durability Deterioration of Concrete under Marine Environment from Material to Structure: A Critical Review. J. Build. Eng..

[5] Sandanayake M., Bouras Y., Haigh R., Vrcelj Z. (2020). Current Sustainable Trends of Using Waste Materials in Concrete—A Decade Review. Sustainability.

[6] Li Y., Zhao S., Dong B., Zhang Y., Zhang Y., Yang J. (2026). Self-Sensing Cementitious Composites Enabled by Geometry-Based Conductive Polymer Reinforcement. Constr. Build. Mater..

[7] Zhang P., Kang L., Wang J., Guo J., Hu S., Ling Y. (2020). Mechanical Properties and Explosive Spalling Behavior of Steel-Fiber-Reinforced Concrete Exposed to High Temperature—A Review. Appl. Sci..

[8] Luhar S., Nicolaides D., Luhar I. (2021). Fire Resistance Behaviour of Geopolymer Concrete: An Overview. Buildings.

[9] Alhamad A., Yehia S., Lublóy É., Elchalakani M. (2022). Performance of Different Concrete Types Exposed to Elevated Temperatures: A Review. Materials.

[10] Ali H.K., Abid S.R., Tayşi N. (2023). Thermal Behaviour and Microstructure of Self-Cured High-Strength Plain and Fibrous Geopolymer Concrete Exposed to Various Fire Scenarios. Buildings.

[11] Ma Q., Guo R., Zhao Z., Lin Z., He K. (2015). Mechanical Properties of Concrete at High Temperature—A Review. Constr. Build. Mater..

[12] Li Y., Liu Z., Jiang J. (2024). Microstructure and Macroscopic Properties of Low-Carbon Concrete Subjected to Elevated Temperature: State-of-the-Art Review. J. Build. Eng..

[13] Manzoor T., Bhat J.A., Shah A.H. (2024). Performance of Geopolymer Concrete at Elevated Temperature—A Critical Review. Constr. Build. Mater..

[14] Hassan A., Arif M., Shariq M., Alomayri T., Pereira S. (2023). Fire Resistance Characteristics of Geopolymer Concrete for Environmental Sustainability: A Review of Thermal, Mechanical and Microstructure Properties. Environ. Dev. Sustain..

[15] Biradar G., Ramanna N., Madduru S.R.C. (2024). An In-Depth Examination of Fire-Related Damages in Reinforced Concrete Structures-A Review. J. Build. Pathol. Rehabil..

[16] Dong B., Yu Y., Feng Y., Yang J., Zhao G., Gao W. (2024). Chloride Ingress Analysis on Alkali-Activated Slag Concrete: A Validated Modelling Framework for Long-Term Assessment. J. Build. Eng..

[17] Chen J., Duan J., Zhu Q., Jin W., Lu C., Lv M., Li W., Li Y., Lv Y. (2025). High Temperature Resistance of Slag/Fly Ash-Based Geopolymer Concrete with Fully Recycled Coarse Aggregate. Constr. Build. Mater..

[18] Hager I., Sitarz M., Mróz K. (2021). Fly-Ash Based Geopolymer Mortar for High-Temperature Application—Effect of Slag Addition. J. Clean. Prod..

[19] Singh N.B., Middendorf B. (2020). Geopolymers as an Alternative to Portland Cement: An Overview. Constr. Build. Mater..

[20] Jwaida Z., Dulaimi A., Mashaan N., Othuman Mydin M.A. (2023). Geopolymers: The Green Alternative to Traditional Materials for Engineering Applications. Infrastructures.

[21] Horan C., Genedy M., Juenger M., van Oort E. (2022). Fly Ash-Based Geopolymers as Lower Carbon Footprint Alternatives to Portland Cement for Well Cementing Applications. Energies.

[22] Kaya M. (2022). Effect of Steel Fiber Additive on High Temperature Resistance in Geopolymer Mortars. Iran. J. Sci. Technol. Trans. Civ. Eng..

[23] Arjun Raj P.K., Sarath D., Nagarajan P., Shashikala A.P. (2023). Behaviour of Geopolymer Concrete at Elevated Temperature- a Comprehensive Review. Mater. Today Proc..

[24] Nodehi M. (2021). A Comparative Review on Foam-Based versus Lightweight Aggregate-Based Alkali-Activated Materials and Geopolymer. Innov. Infrastruct. Solut..

[25] Paswan R.K., Kumar P., Kumar V., Sembeta R.Y. (2025). Mechanical Properties of Alkali Activated Slag Binder Based Concrete at Elevated Temperatures. Discov. Sustain..

[26] Sun H., Zhang Z., Liang Y., Ji T. (2025). Preparation and Fireproof Performance of Alkali Activated Cement Based Fire Resistive Coatings with Different Alkali Activators for Steel Structures. Case Stud. Constr. Mater..

[27] Abdelmelek N., Lubloy E. (2021). Evaluation of the Mechanical Properties of High-Strength Cement Paste at Elevated Temperatures Using Metakaolin. J. Therm. Anal. Calorim..

[28] Luccioni B.M., Figueroa M.I., Danesi R.F. (2003). Thermo-Mechanic Model for Concrete Exposed to Elevated Temperatures. Eng. Struct..

[29] Pasztetnik M., Wróblewski R. (2021). A Literature Review of Concrete Ability to Sustain Strength after Fire Exposure Based on the Heat Accumulation Factor. Materials.

[30] Suh H., Jee H., Kim J., Kitagaki R., Ohki S., Woo S., Jeong K., Bae S. (2020). Influences of Rehydration Conditions on the Mechanical and Atomic Structural Recovery Characteristics of Portland Cement Paste Exposed to Elevated Temperatures. Constr. Build. Mater..

[31] Xu L., Hu X., Tang R., Zhang X., Xia Y., Ran B., Liu J., Zhuang S., Tian W. (2025). Microscopic Mechanical Properties and Physicochemical Changes of Cement Paste Exposed to Elevated Temperatures and Subsequent Rehydration. Materials.

[32] Amiri M., Aryanpour M., Porhonar F. (2022). Microstructural Study of Concrete Performance after Exposure to Elevated Temperatures via Considering C–S–H Nanostructure Changes. High Temp. Mater. Process..

[33] Alnadish A.M. (2026). Geopolymer Concrete: A Comprehensive Review of Current Research Trends, Challenges, and Environmental-Economic Implications. Arch. Comput. Methods Eng..

[34] Mohan Kumar S.G.K., Kinuthia J.M., Oti J., Adeleke B.O. (2025). Geopolymer Chemistry and Composition: A Comprehensive Review of Synthesis, Reaction Mechanisms, and Material Properties—Oriented with Sustainable Construction. Materials.

[35] Ralli Z.G., Pantazopoulou S.J. (2020). State of the Art on Geopolymer Concrete. Int. J. Struct. Integr..

[36] Olayinka R., Jafari R., Fiset M. (2025). Shrinkage Characteristics of Geopolymer Concrete: A Comprehensive Review. Materials.

[37] Barbhuiya S., Das B.B., Kapoor K., Davis R., Kondraivendhan B. (2026). Fire Resistance and Thermal Stability of Geopolymer Concrete. Discov. Concr. Cem..

[38] Jiang X., Zhang Y., Zhang Y., Ma J., Xiao R., Guo F., Bai Y., Huang B. (2023). Influence of Size Effect on the Properties of Slag and Waste Glass-Based Geopolymer Paste. J. Clean. Prod..

[39] Yuan W., Li H., Zhang D., Wang Q., Yan Z. (2025). Effects of the Physicochemical Properties of Sodium Metasilicate on the Hydration Kinetics and High-Temperature Stability of One-Part Alkali-Activated Fly Ash-Slag Materials. Constr. Build. Mater..

[40] Cai C., Zhang Z., Shi C., Zhao J., Kong J., Li C., Rodriguez E.D., She W., Wang H., Jiang Z. (2026). Synthesis of Novel Tuff-Metakaolin Blended Based Geopolymers: Thermal Performance and Self-Foaming at 1200 °C. Cem. Concr. Compos..

[41] Shubaili M. (2026). Effect of GGBFS and Fly Ash on Elevated Temperature Resistance of Pumice-Based Geopolymers. Infrastructures.

[42] Win T.T., Panwisawas C., Jongvivatsakul P., Pansuk W., Prasittisopin L. (2023). Effects of Fly Ash Composition to Mitigate Conversion of Calcium Aluminate Cement Composites. Buildings.

[43] Win T.T., Prasittisopin L., Jongvivatsakul P., Likitlersuang S. (2024). Investigating the Role of Steel and Polypropylene Fibers for Enhancing Mechanical Properties and Microstructural Performance in Mitigating Conversion Effects in Calcium Aluminate Cement. Constr. Build. Mater..

[44] Lee J.-M., Yang H.J., Jang I.-Y., Kim S.K., Jung D.-H. (2022). Comparison of Calcium Aluminate Cements on Hydration and Strength Development at Different Initial Curing Regimes. Case Stud. Constr. Mater..

[45] Şengül K., Erdoğan S.T. (2021). Influence of Ground Perlite on the Hydration and Strength Development of Calcium Aluminate Cement Mortars. Constr. Build. Mater..

[46] Eren F., Keskinateş M., Felekoğlu B., Tosun Felekoğlu K. (2022). The Role of Pre-Heating and Mineral Additive Modification on Long-Term Strength Development of Calcium Aluminate Cement Mortars. Constr. Build. Mater..

[47] Liu C., Chen J. (2022). High Temperature Degradation Mechanism of Concrete with Plastering Layer. Materials.

[48] Kiran T., Yadav S.K., N A., Mathews M.E., Andrushia D., Lubloy E., Kodur V. (2022). Performance Evaluation of Lightweight Insulating Plaster for Enhancing the Fire Endurance of High Strength Structural Concrete. J. Build. Eng..

[49] Kanagaraj B., Anand N., Jerry R., Samuvel Raj R., Andrushia D., Lubloy E. (2023). Influence of Protective Coating on Flexural Behaviour of High Strength Self-Compacting Geopolymer Concrete Beams Exposed to Standard Fire Temperature. Case Stud. Constr. Mater..

[50] (2024). Standard Test Method for Compressive Strength of Hydraulic Cement Mortars (Using 2-in. or [50-Mm] Cube Specimens).

[51] (2022). Standard Test Method for Pulse Velocity Through Concrete.

[52] (2021). Standard Test Method for Density, Absorption, and Voids in Hardened Concrete.

[53] Hu X., Wen B., Gao G., Niu D., Miao J. (2026). Experimental Study on Bond Performance of Steel Bars and Coal Gangue Concrete after High Temperatures. J. Build. Eng..

[54] Yao X., Han Y., Shen L., Zhu D. (2024). Experimental Study on the Effect of Polypropylene Fiber on Compressive Strength and Fracture Properties of High-Strength Concrete after Elevated Temperatures. J. Build. Eng..

[55] Sadrmomtazi A., Gashti S.H., Tahmouresi B. (2020). Residual Strength and Microstructure of Fiber Reinforced Self-Compacting Concrete Exposed to High Temperatures. Constr. Build. Mater..

[56] Abdelmelek N., Lubloy E. (2022). The Impact of Metakaolin, Silica Fume and Fly Ash on the Temperature Resistance of High Strength Cement Paste. J. Therm. Anal. Calorim..

[57] Kim T., Kim W., Im H., Lee T. (2026). Residual Mechanical and Structural Properties of Non-Calcined Hwangto Concrete After Exposure to High Temperatures. Materials.

[58] Demirboğa R., Türkmen İ., Farhan K.Z., Bingöl A.F., Tortum A., Alymani A.A. (2026). Elevated-Temperature Performance of Concrete with Expanded Perlite Fine Aggregate: Experimental and ANN Analysis. Front. Built Environ..

[59] Zhang T., Zhang M., Chen Q., Zhu H., Yan Z. (2023). Enhancing the Thermo-Mechanical Properties of Calcium Aluminate Concrete at Elevated Temperatures Using Synergistic Flame-Retardant Polymer Fibres. Cem. Concr. Compos..

[60] Salli Bideci Ö., Yılmaz H., Gencel O., Bideci A., Çomak B., Nodehi M., Ozbakkaloglu T. (2023). Fiber-Reinforced Lightweight Calcium Aluminate Cement-Based Concrete: Effect of Exposure to Elevated Temperatures. Sustainability.

[61] Yu C., Huang B., Cai X., Wang Y., Ye C., Liao R. (2026). Comparative Effectiveness and Mechanisms of Different Cement Types in Solidifying/Stabilizing Copper Ions. J. Environ. Manag..

[62] Yang H.J., Jin S.H., Ann K.Y. (2016). Chloride Transport of High Alumina Cement Mortar Exposed to a Saline Solution. Adv. Mater. Sci. Eng..

[63] Tang J., Ma W., Gu Z., Zhang Y., Fang D., Zhao L. (2023). Study on Mechanical Properties and Microstructure of Aluminate Cement-Based Materials Incorporating Recycled Brick Powder after Exposure to Elevated Temperatures. J. Build. Eng..

[64] Amer Salih M., Farzadnia N., Demirboga R., Ali A.A.A. (2022). Effect of Elevated Temperatures on Mechanical and Microstructural Properties of Alkali-Activated Mortar Made up of POFA and GGBS. Constr. Build. Mater..

[65] Pratap B., Paswan R.K., Kumar P. (2025). Mechanical and Micro-Characterization of Alkali-Activated Concrete under High-Temperature Exposure. Struct. Concr..

[66] Fu B., Zhu M., Dong H., Meng F. (2026). Study on the Properties of High-Strength Slag-Fly Ash-Based Geopolymer Concrete After Exposure to Elevated Temperatures. Sustainability.

[67] Amin M., Heniegal A.M., Zeyad A.M., Agwa I.S., Fattouh M.S. (2026). Performance-Based Comparison of UHPC and UHPGC Incorporating FA, GBFS, and MK Under Ambient and Elevated Temperatures. Iran. J. Sci. Technol. Trans. Civ. Eng..

[68] Kadhim S., Çevik A., Niş A., Bakbak D., Aljanabi M. (2022). Mechanical Behavior of Fiber Reinforced Slag-Based Geopolymer Mortars Incorporating Artificial Lightweight Aggregate Exposed to Elevated Temperatures. Constr. Build. Mater..

[69] Natarajan P., Sivasakthi M., Revathi T., Jeyalakshmi R. (2021). A Comparative Study on Fly Ash Pozzolanic Cement Mortar and Ambient-Cured Alkali-Activated Fly Ash–GGBS Cement Mortar after Exposure to Elevated Temperature. Innov. Infrastruct. Solut..

[70] Hosan A., Haque S., Shaikh F. (2016). Compressive Behaviour of Sodium and Potassium Activators Synthetized Fly Ash Geopolymer at Elevated Temperatures: A Comparative Study. J. Build. Eng..

[71] Saludung A., Azeyanagi T., Ogawa Y., Kawai K. (2023). Mechanical and Microstructural Evolutions of Fly Ash/Slag-Based Geopolymer at High Temperatures: Effect of Curing Conditions. Ceram. Int..

[72] Shdeifat H., Kalfat R., Al-Mahaidi R. (2026). Thermomechanical Performance of Ambient-Cured Fly Ash Geopolymers Under Fire Exposure: Role of Activator Type and Mix Design. Buildings.

[73] Kim W., Jeong K., Choi H., Lee T. (2022). Correlation Analysis of Ultrasonic Pulse Velocity and Mechanical Properties of Normal Aggregate and Lightweight Aggregate Concretes in 30–60 MPa Range. Materials.

[74] Shaladi R.J., Johari M.A.M., Ahmad Z.A., Mijarsh M.J.A. (2022). The Influence of Palm Oil Fuel Ash Heat Treatment on the Strength Activity, Porosity, and Water Absorption of Cement Mortar. Environ. Sci. Pollut. Res..

[75] Karimaei M., Dabbaghi F., Dehestani M., Rashidi M. (2021). Estimating Compressive Strength of Concrete Containing Untreated Coal Waste Aggregates Using Ultrasonic Pulse Velocity. Materials.

[76] Srivastava A., Singh S.K., Sharma C.S. (2021). Correlation Between Ultrasonic Pulse Velocity (UPV) and Compressive Strength of Coal Bottom Ash Mortar. J. Inst. Eng. India Ser. A.

[77] Ezzedine El Dandachy M., Hassoun L., El-Mir A., Khatib J.M. (2024). Effect of Elevated Temperatures on Compressive Strength, Ultrasonic Pulse Velocity, and Transfer Properties of Metakaolin-Based Geopolymer Mortars. Buildings.

[78] Santos E.W.J., Dourado T.C., Costa-Félix R.P.B. (2026). Metrological Ultrasonic Evaluation for Hierarchical Classification of Fire-Induced Thermal Damage in Concrete. Measurement.

[79] Benjeddou O., Katman H.Y., Jedidi M., Mashaan N. (2022). Experimental Investigation of the High Temperatures Effects on Self-Compacting Concrete Properties. Buildings.

[80] Shariq M., Ahmad F., Masood A., Hashmi A.F., Ayaz M. (2025). Development of Novel Concrete with Sustainable Waste Materials: A Comprehensive Study on Strength, Pulse Velocity, and Rebound Hammer Number at Elevated Temperatures Using Predictive Models. Constr. Build. Mater..

[81] Kim W., Choi H., Lee T. (2023). Investigating Ultrasonic Pulse Velocity Method for Evaluating High-Temperature Properties of Non-Sintered Hwangto-Mixed Concrete as a Cement Replacement Material. Materials.

[82] Lima H.M., Fernandes Neto J.A.D., Haach V.G., Munaiar Neto J. (2025). Physical and Mechanical Properties of Cement-Lime Mortar for Masonry at Elevated Temperatures: Destructive and Ultrasonic Testing. J. Build. Eng..

[83] Kim W., Choi H., Lee T. (2023). Residual Compressive Strength Prediction Model for Concrete Subject to High Temperatures Using Ultrasonic Pulse Velocity. Materials.

[84] Wang Y., Cui J., Deng J., Zhou H. (2023). Experimental Study of Thermally Damaged Concrete under a Hygrothermal Environment by Using a Combined Infrared Thermal Imaging and Ultrasonic Pulse Velocity Method. Materials.

[85] Fadiel A.A.M., Abu-Lebdeh T., Munteanu I.S., Niculae E., Petrescu F.I.T. (2023). Mechanical Properties of Rubberized Concrete at Elevated Temperatures. J. Compos. Sci..

[86] Zhang G.-Z., Zhang S.-Z., Shi Y.-M., Liu J.-Z., Sun F., Wang X.-Y. (2026). Light-Responsive Algal-Bacterial Symbiosis for Crack Self-Healing in Cement Mortar: Evidence of Surface-to-Depth Healing Mismatch. Cem. Concr. Compos..

[87] Islam M.Z., Sohel K.M.A., Al-Jabri K., Al Harthy A. (2021). Properties of Concrete with Ferrochrome Slag as a Fine Aggregate at Elevated Temperatures. Case Stud. Constr. Mater..

[88] Mohammed A. (2021). Abed Indirect Evaluation of the Compressive Strength of Recycled Aggregate Concrete at Long Ages and after Exposure to Freezing or Elevated Temperatures. Russ. J. Nondestruct. Test..

[89] Wu D., Mao Z., Zhang J., Li S., Ma Q. (2023). Performance Evaluation of Concrete with Waste Glass after Elevated Temperatures. Constr. Build. Mater..

[90] Yıldırım M., Özhan H.B. (2023). Durability Properties of Basalt Fiber-Reinforced Mortars with Different Mineral Admixtures Exposed to High Temperatures. Constr. Build. Mater..

[91] Vafaei D., Ma X., Hassanli R., Duan J., Zhuge Y. (2022). Microstructural and Mechanical Properties of Fiber-Reinforced Seawater Sea-Sand Concrete under Elevated Temperatures. J. Build. Eng..

[92] Hachemi S., Khattab M., Benzetta H. (2023). Enhancing the Performance of Concrete after Exposure to High Temperature by Coarse and Fine Waste Fire Brick: An Experimental Study. Constr. Build. Mater..

[93] Tiwary A.K., Singh S., Kumar R., Chohan J.S., Sharma S., Singh J., Li C., Ilyas R.A., Asyraf M.R.M., Malik M.A. (2022). Effects of Elevated Temperature on the Residual Behavior of Concrete Containing Marble Dust and Foundry Sand. Materials.

[94] Ankit, Tiwary A.K., Hasrat H.A. (2025). Influence of Zirconia and Rice Husk Ash on Residual Characteristics of Sustainable Cement Mortar after Exposure to Elevated Temperatures. Discov. Mater..

[95] Zhou J., Lu D., Yang Y., Gong Y., Ma X., Yu B., Yan B. (2020). Physical and Mechanical Properties of High-Strength Concrete Modified with Supplementary Cementitious Materials after Exposure to Elevated Temperature up to 1000 °C. Materials.

[96] Kunthawatwong R., Wongsa A., Ekprasert J., Sukontasukkul P., Sata V., Chindaprasirt P. (2023). Performance of Geopolymer Mortar Containing PVC Plastic Waste from Bottle Labels at Normal and Elevated Temperatures. Buildings.

[97] Li Q., Li H., Liang T. (2023). Effect of Non-Intumescent Fireproof Coating on the Durability of Concrete Exposed to Elevated Temperature. Constr. Build. Mater..

[98] Quadri A.I., Bankole A.O. (2024). The Effect of Elevated Temperature on the Performance of Pozzolanic Cement Mortar. Discov. Civ. Eng..

[99] Praneedpolkrang P., Chaiwasee N., Koedmontree P., Suthiwong A., Kaur H., Jaturapitakkul C., Tangchirapat W. (2024). Effects of Elevated Temperature on Mechanical Properties and Microstructures of Alkali-Activated Mortar Made from Low Calcium Fly Ash-Calcium Carbide Residue Mixture. Case Stud. Constr. Mater..

[100] Mohamad S.A., Al-Hamd R.K.S., Khaled T.T. (2020). Investigating the Effect of Elevated Temperatures on the Properties of Mortar Produced with Volcanic Ash. Innov. Infrastruct. Solut..

[101] Al-Ameri R.A., Abid S.R., Murali G., Ali S.H., Özakça M. (2021). Residual Repeated Impact Strength of Concrete Exposed to Elevated Temperatures. Crystals.

[102] Çelikten S., Baran B. (2024). A Comprehensive Assessment of Mechanical and Environmental Properties of Waste Basalt Powder-Modified High Strength Mortars Exposed to High Temperature. Next Mater..

[103] Zhao J., Wang K., Wang S., Wang Z., Yang Z., Shumuye E.D., Gong X. (2021). Effect of Elevated Temperature on Mechanical Properties of High-Volume Fly Ash-Based Geopolymer Concrete, Mortar and Paste Cured at Room Temperature. Polymers.

[104] Abed A.-T., Éva L. (2026). High-Temperature Behavior of Expanded-Perlite-Modified Cement Mortars: An Experimental Investigation. Constr. Build. Mater..

[105] Pereira M.C., Soares A., Flores-Colen I., Correia J.R. (2020). Influence of Exposure to Elevated Temperatures on the Physical and Mechanical Properties of Cementitious Thermal Mortars. Appl. Sci..

[106] Jiang X., Xiao R., Zhang M., Hu W., Bai Y., Huang B. (2020). A Laboratory Investigation of Steel to Fly Ash-Based Geopolymer Paste Bonding Behavior after Exposure to Elevated Temperatures. Constr. Build. Mater..

[107] Ahsan M.H., Siddique M.S., Farooq S.H., Usman M., Ul Aleem M.A., Hussain M., Hanif A. (2022). Mechanical Behavior of High-Strength Concrete Incorporating Seashell Powder at Elevated Temperatures. J. Build. Eng..

[108] Afzal M.T., Khushnood R.A. (2021). Influence of Carbon Nano Fibers (CNF) on the Performance of High Strength Concrete Exposed to Elevated Temperatures. Constr. Build. Mater..

[109] Ahmad F., Rawat S., Yang R., Zhang L., Zhang Y.X. (2025). Fire Resistance and Thermal Performance of Hybrid Fibre-Reinforced Magnesium Oxychloride Cement-Based Composites. Constr. Build. Mater..

[110] Palizi S., Toufigh V. (2023). Fire-Induced Damage Assessment of Cementless Alkali-Activated Slag-Based Concrete. Constr. Build. Mater..

[111] Günel G., Alakara E.H., Demir I., Sevim O. (2025). Thermal Behavior and Mechanical Performance of Glass Waste-Based Geopolymer Composites: Influence of Elevated Temperatures and Cooling Regimes. Constr. Build. Mater..

[112] Boquera L., Castro J.R., Pisello A.L., Fabiani C., D’Alessandro A., Ubertini F., Cabeza L.F. (2022). Effect of the Curing Process on the Thermomechanical Properties of Calcium Aluminate Cement Paste under Thermal Cycling at High Temperatures for Thermal Energy Storage Applications. J. Energy Storage.

[113] Kuri J.C., Majhi S., Sarker P.K., Mukherjee A. (2021). Microstructural and Non-Destructive Investigation of the Effect of High Temperature Exposure on Ground Ferronickel Slag Blended Fly Ash Geopolymer Mortars. J. Build. Eng..

[114] Chen H.-J., Yu Y.-L., Tang C.-W. (2020). Mechanical Properties of Ultra-High Performance Concrete before and after Exposure to High Temperatures. Materials.

[115] Tahwia A.M., Ellatief M.A., Bassioni G., Heniegal A.M., Elrahman M.A. (2023). Influence of High Temperature Exposure on Compressive Strength and Microstructure of Ultra-High Performance Geopolymer Concrete with Waste Glass and Ceramic. J. Mater. Res. Technol..

[116] Nikbin I.M., Mehdipour S., Dezhampanah S., Mohammadi R., Mohebbi R., Moghadam H.H., Sadrmomtazi A. (2020). Effect of High Temperature on Mechanical and Gamma Ray Shielding Properties of Concrete Containing Nano-TiO2. Radiat. Phys. Chem..

[117] Meftah H., Arabi N. (2024). Effects of Elevated Temperatures’ Exposure on the Properties of Concrete Incorporating Recycled Concrete Aggregates. Constr. Build. Mater..

[118] Doğruyol M., Ayhan E., Karaşin A. (2024). Effect of Waste Steel Fiber Use on Concrete Behavior at High Temperature. Case Stud. Constr. Mater..

[119] Ali A.E., Faried A.S., Osman K.M. (2024). Effect of Elevated Temperature on Properties of Concrete Containing Different Types of Nano-Materials. Constr. Build. Mater..

[120] Wang W., Liu X., Guo L., Duan P. (2019). Evaluation of Properties and Microstructure of Cement Paste Blended with Metakaolin Subjected to High Temperatures. Materials.

